# Twelve New Species Reveal Cryptic Diversification in Foliicolous Lichens of *Strigula* s.lat. (*Strigulales*, *Ascomycota*)

**DOI:** 10.3390/jof8010002

**Published:** 2021-12-21

**Authors:** Shu-Hua Jiang, Robert Lücking, Hua-Jie Liu, Xin-Li Wei, Amanda Barreto Xavier-Leite, Carlos Viñas Portilla, Qiang Ren, Jiang-Chun Wei

**Affiliations:** 1 State Key Laboratory of Mycology, Institute of Microbiology, Chinese Academy of Sciences, Beijing 100101, China; weixl@im.ac.cn (X.-L.W.); renqiang@im.ac.cn (Q.R.); 2 Botanischer Garten und Botanisches Museum, Freie Universität Berlin, Königin-Luise-Straße 6–8, 14195 Berlin, Germany; r.luecking@bgbm.org; 3 College of Life Sciences, Institute of Life Science and Green Development, Hebei University, Baoding 071002, China; liuhuajie@foxmail.com; 4 University of Chinese Academy of Sciences, Beijing 100049, China; 5 Programa de Pós-Graduação em Sistemática e Evolução, CB, Universidade Federal do Rio Grande do Norte, Campus Universitário, Natal 59072-970, RN, Brazil; amandabxleite@gmail.com; 6 Jardín Botánico Nacional, Universidad de La Habana, Carretera El Rocío km 3½, Calabazar, Boyeros, La Habana C.P. 19230, Cuba; carlosvip@fbio.uh.cu

**Keywords:** diversity, morphology, new taxa, *Strigulaceae*, systematics

## Abstract

We employed a molecular phylogenetic approach using five markers (ITS, nuSSU, nuLSU, TEF1-α, and RPB2) to assess potential cryptic speciation in foliicolous members of *Strigula* s.lat. (*Strigulaceae*), including the recently segregated genera *Phylloporis*, *Puiggariella*, *Raciborskiella*, *Racoplaca*, and *Serusiauxiella*, from tropical areas in Asia, with selected materials from the Neotropics as reference. On the basis of combined molecular and phenotypic datasets, two new species of *Racoplaca* and 10 new species of *Strigula* s.str. are described: *Racoplaca macrospora* sp. nov., *R. maculatoides* sp. nov., *Strigula guangdongensis* sp. nov., *S. intermedia* sp. nov., *S. laevis* sp. nov., *S. microcarpa* sp. nov., *S. pseudoantillarum* sp. nov., *S. pseudosubtilissima* sp. nov., *S. pycnoradians* sp. nov., *S. sinoconcreta* sp. nov., *S. stenoloba* sp. nov., and *S. subtilissimoides* sp. nov. In addition, we propose the new combination *Phylloporis palmae* comb. nov. (≡ =*Manaustrum palmae*) and we validate the earlier combination *Racoplaca melanobapha* comb. nov. (≡ *Verrucaria melanobapha*; *Strigula melanobapha*). Our data clearly indicate a considerable degree of cryptic diversification in foliicolous representatives of *Strigula* s.lat., particularly in the presumably widespread taxa *Strigula antillarum*, *S. concreta*, *S. nitidula*, and *S. smaragdula*. Given that these phylogenetic revisions are thus far limited to few regions, we predict that our findings only represent the proverbial tip of the iceberg in this group of lichenized fungi.

## 1. Introduction

Understanding species delimitation is crucial for biological and applied sciences and for conservation assessments [1,2]. In lichenised fungi, alpha-taxonomy has traditionally been based on morphological, anatomical, and/or chemical characters [3,4]. However, ontogenetic, epigenetic, and environmental factors may influence phenotypic features and, as a result, it is often difficult to distinguish phylogenetically informative characters from intraspecific variability [5,6,7,8]. In addition, evolutionary homoplasy is frequent in fungi, including lichens, often leading to phenotypically cryptic, albeit not directly related species [9,10].

Integrative taxonomic studies based on molecular and morphological datasets could serve to re-evaluate earlier classifications and provide more accurate species-level delimitations [11]. Such studies have been performed in many different lichen groups, including *Cora* [12], *Leptogium* [13], *Lobariella* [14], *Parmelia* [15,16,17], *Parmelina* [18], *Peltigera* [19], *Physconia* [20,21], *Pseudocyphellaria* [22,23], *Rhizoplaca* [24], *Sticta* [25,26], and *Usnea* [27]. In many cases, subtle, previously overlooked, or underestimated phenotypic characters would support the distinction of newly recognised species. However, phylogenetically recognised species may also be morphologically cryptic, for example, in complexes such as *Caloplaca crenulatella* [28], *Cladia aggregata* [29], *Graphis scripta* [30], *Melanelixia glabra* [31], *Parmelina tiliacea* [32], *Porina epiphylla* and *P*. *rufula* [33], *Punctelia rudecta* [34], and *Rhizoplaca melanophthalma* [35]. Here, we follow Lücking et al. [36] in considering species cryptic if there are no clearly discernable phenotypic diagnostic characters and near-cryptic if such characters are subtle and require careful analysis to be properly assessed.

A large proportion of undescribed fungal species richness is expected to be found in poorly studied areas, such as tropical forests [9,10,37,38]. Foliicolous representatives of the lichenised family *Strigulaceae* (*Strigulales*, *Dothideomycetes*, *Ascomycota*) [39,40,41] are widespread in the tropics and subtropics [42,43,44,45]. Five foliicolous genera (*Flavobathelium*, *Oletheriostrigula*, *Phyllobathelium*, *Phyllocratera*, and *Strigula*) were previously accepted in the family [40,46], but a phylogenetic revision of foliicolous taxa in *Strigula* s.lat. triggered the resurrection of the genera *Phylloporis*, *Puiggariella*, *Raciborskiella*, and *Racoplaca*, and the description of a new genus, *Serusiauxiella* [47]. Many species in these genera are presumably widespread, often considered pantropical or even subcosmopolitan. The most prominent example is *Strigula smaragdula*, originally described from Nepal, for which Santesson (1952) [42], under the name *S. elegans*, adopted a broad concept, including even populations from temperate Europe. While the name *Strigula buxi* has been reinstated for the European lineage [44], recent phylogenetic studies have shown that, even in (sub)tropical Asia, *S. smaragdula* s.lat. is a collective taxon, with a considerable degree of cryptic or near-cryptic speciation [48,49,50]. Unfortunately, the small size of the thalli and the tendency to grow intermingled with other foliicolous lichens and nonlichenised fungi, typically below the leaf cuticle, poses a challenge to molecular phylogenetic approaches. In addition, the DNA of foliicolous lichens appears to degrade rapidly, making it necessary to work with relatively fresh collections. 

Here, we report the results from a careful study of numerous new collections of foliicolous *Strigula* s.lat. from Asia, and Central and South America. To assess the traditional alpha-taxonomy in foliicolous representatives of *Strigula* s.lat. based on phenotype characters, we targeted various species complexes, employing a multi-marker phylogeny (ITS, nuSSU, nuLSU, TEF1-α, and RPB2) under maximum likelihood and Bayesian frameworks.

## 2. Materials and Methods

### 2.1. Material Examined

Newly collected or previously frozen collections were examined from Asia (Cambodia, China, and Thailand), and Central and South America (Brazil, Costa Rica, Cuba, Guatemala, and Panama). Specimens are preserved in the Fungarium-Lichenum of the Institute of Microbiology, Chinese Academy of Sciences (HMAS–L) and the herbarium of the Botanischer Garten und Botanisches Museum of the Freie Universität Berlin (B). 

### 2.2. Phenotypic Analyses

A LEICA M125 dissecting microscope (LEICA Microsystems, Singapore) was used for the morphological studies and a ZEISS Axioscope 2 (ZEISS, Göttingen, Germany) compound microscope for the anatomical observations and measurements. Photographs were made with an AxioCam MRc5 connected to a ZEISS Imager A2-M2 microscope (ZEISS, Göttingen, Germany). Thin-layer chromatography (TLC) was employed for the detection of lichen substances with solvent system C [51].

### 2.3. Phylogenetic Analyses

#### 2.3.1. DNA Extraction and PCR Amplification

A total of 174 specimens were subjected to DNA extraction (Table S1), for which a modified CTAB method [52] was used. Extracted DNA, suspended in ddH_2_O, was amplified through polymerase chain reaction (PCR). The nuclear ribosomal RNA gene region, including the internal transcribed spacer (ITS), was amplified using the primer pair ITS5 and ITS4 [53]. Partial nuSSU sequences were amplified and sequenced using combinations of the primers nu-SSU-0402-5′/NS19UCB [54], nu-SSU-0852-3′/NS20UCB [54], f-nu-SSU-0176-5′-mpn [55], f-nu-SSU-0801-3′-mpn [55], SF5 [56], and SR5 [56]. A portion of the fungal nuclear ribosomal large subunit nuLSU was amplified and sequenced using the primers ITS3 [53] and LR72 [56]. Partial TEF1-α sequences were generated using the primers TEF1a-983 F [57] and TEF1a-1567R-HTL [55]. The second largest subunit of the RNA polymerase II, RPB2, was amplified and sequenced using the primers fRPB2-5F and fRPB2-7cR [58]. 

PCR reactions were carried out in 25 μL reaction volumes and the components used were: 2 μL total DNA, 1 μL each primer (10 μM), 12.5 μL 2 × Taq MasterMix, and 8.5 μL ddH_2_O. Amplification was performed using a Biometra T-Gradient thermal cycler. Cycling parameters for nuLSU, ITS, and nuSSU were set to an initial denaturation at 95 °C for 5 min, followed by 35 cycles of denaturation at 94 °C for 30 s, annealing at 54 °C for 30 s, extension at 72 °C for 1 min, and a final extension at 72 °C for 10 min. PCR amplifications of TEF1-α were initiated with a 2 min denaturation at 94 °C. The annealing temperature in the first amplification cycle was 66 °C, which was subsequently incrementally reduced by 1 °C per cycle over the next 9 cycles. An additional 30 amplification cycles were then performed, each consisting of 30 s denaturation at 94 °C, a 30 s annealing step at 56 °C, and a 1 min extension at 72 °C, concluding with a 10 min incubation at 72 °C [57]. The PCR conditions of RPB2 included: initial denaturation at 95 °C for 5 min; 35 cycles of 1 min at 95 °C, 2 min at 50 °C, an increase of 1 °C/5 s to 72 °C, and 2 min at 72 °C; and a 10-min incubation at 72 °C [58]. PCR products were checked on 0.8% agarose electrophoresis gels stained with ethidium bromide, and then sent to Majorbiology (Changping District, Beijing, China) for sequencing.

#### 2.3.2. Sequence Alignment and Phylogenetic Analyses

Sequences for each marker were joined with others obtained from GenBank (Table S1), generating a separate ITS and a concatenated four-locus (nuSSU, nuLSU, TEF-α, and RPB2) dataset. Each marker was firstly aligned independently with MAFFT v. 7 [59], and the combinability was tested [60]. Each partition was firstly analysed separately using MrBayes. The Markov chain Monte Carlo algorithm of MrBayes ran with 20 chains simultaneously, each initiated with a random tree, for 2 M generations, sampling every 20th generation for a total of 100,000 trees sampled. The first 4500 sampled trees were discarded before calculating the majority-rule consensus tree to ensure that all chains had converged at a single level. A majority-rule consensus tree was calculated with DendroPy v. 4.5.2 for the remaining 95500 B/MCMC sampled trees [61]. The majority-rule consensus tree of each gene was constructed through SumTrees with the parameters “summary-target = consensus --burnin = 4500 --support-as-labels --min-clade-freq = 0.1”. The conflict was assumed to be significant if different relationships for the same set of taxa (one being monophyletic and the other being nonmonophyletic), all with posterior probabilities (PP) 99%, were observed on the majority-rule consensus trees. Only if no significant conflict was detected throughout the majority-rule consensus trees, using this criterion, would the four partitions be combined [60].

An ML tree involving 1000 pseudoreplicates was generated by IQ-TREE v1.6.6 [62]. For this analysis, the best-fit substitution model was selected using ModelFinder [63]. GTR + F + I + G4 was selected as the best model for the ITS, and TIM2 + F + I + G4 for the four-locus dataset.

Bayesian analysis was performed in MrBAYES [64] under the general time reversible model, including estimation of invariant sites and a discrete gamma distribution with six rate categories (GTR + I + G), both for the single marker and for the combined analyses. A run with 5 M generations and employing 20 simultaneous chains was executed. Posterior probabilities above 95% and a bootstrap support value above 70% were considered threshold values. 

Phylogenetic trees were drawn using FigTree v. 1.4.2 [65]. All new sequences generated in this study were deposited in GenBank and the alignments in TreeBASE (http://purl.org/phylo/treebase/phylows/study/TB2:S27476 (accessed on 12 October 2021)).

#### 2.3.3. Species Tree Assessment

Firstly, we utilised IQ-TREE v1.6.6 [62] to construct an ML phylogenetic gene tree for each fragment (nuSSU, ITS, nuLSU, TEF-α, and RPB2), with 1000 pseudoreplicates, respectively. ASTRAL finds the species tree that has the maximum number of shared induced quartet trees with the set of gene trees, subject to the constraint that the set of bipartitions in the species tree comes from a predefined one. We conducted the analysis of the best supported ML gene trees in the multi-individual version of ASTRAL v5.7.7 [66,67] to estimate a species tree annotated with posterior probabilities, as nodes support.

## 3. Results

### 3.1. Phylogenetic Analyses

**Figure 1:**
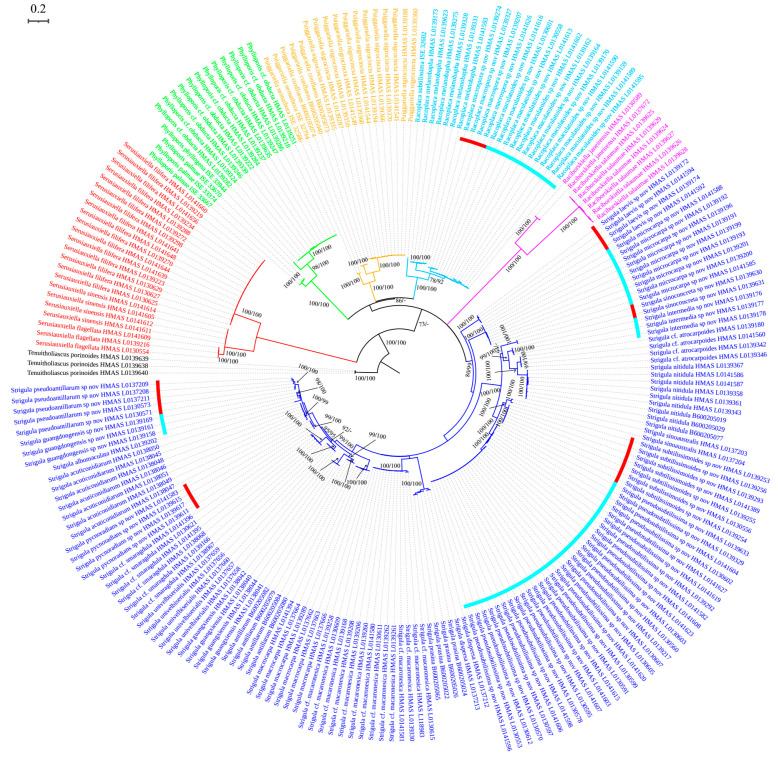
Phylogenetic tree constructed through ML analyses based on 225 ITS sequences with an alignment length of 448 bp. Bootstrap values above 70% (**left**) and Bayesian inference posterior probabilities above 95% (**right**) are given above branches (ML-BS/B-PP). Newly recognised species are marked with red or blue stripes.

The dataset includes 169 ITS sequences, 20 nuLSU sequences, 17 nuSSU sequences, 19 TEF1-α sequences, and 19 RPB2 sequences newly generated for this study. Since we obtained a much larger number of ITS sequences, this gene locus was analysed separately (Figure 1) and subsequently compared with the concatenated tree based on the other four gene loci.

The ITS tree contained a total of 225 terminals, based on an alignment with a length of 448 bp. The topology (ML tree shown) was consistent with the earlier findings by Jiang et al. [47], recovering the six genera now distinguished within foliicolous *Strigula* s.lat. (Figure 1). Both the genera and almost all species had strong support through ML and Bayesian analyses. The ITS phylogeny revealed 11 new lineages, here introduced as new species, two in *Racoplaca* (*R. maculatoides*, *R. macrospora*) and nine in *Strigula* s.str. (*S. guangdongensis*, *S. intermedia*, *S. laevis*, *S. microcarpa*, *S. pseudoantillarum*, *S. pseudosubtilissima*, *S. pycnoradians*, *S. sinoconcreta*, *S. subtilissimoides*). In addition, the ITS-based phylogeny supported the separation of Brazilian material of *Phylloporis* with a punctate thallus from *P. phyllogena* s.str. (Figure 1); for this material, the name *P. palmae* is taken up below. A further new species of *Strigula*, *S. stenoloba*, is described based on phenotype only, as no sequence data could be generated for this material.

**Figure 2:**
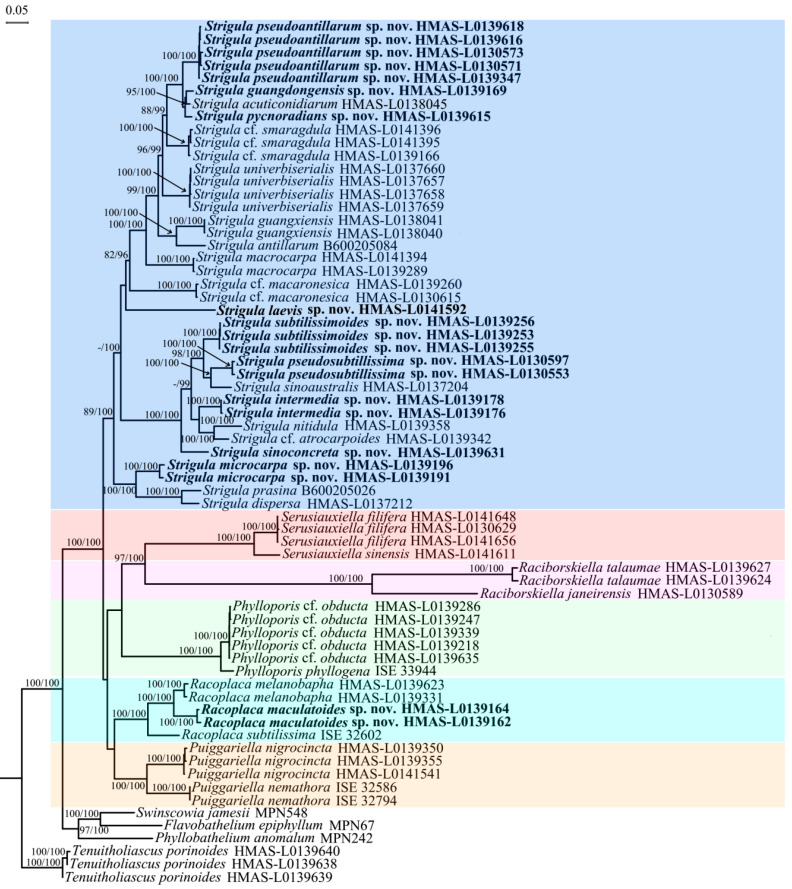
Phylogenetic tree constructed through ML analyses based on four markers (nuSSU, nuLSU, TEF1-α, and RPB2), with a total alignment length of 4373 bp. Maximum likelihood bootstrap support value above 70% (**left**) and Bayesian inference posterior probabilities above 95% (**right**) are shown above branches (ML-BS/B-PP). Terminals in boldface indicate newly generated sequences for this study.

The majority-rule consensus tree sampled with B/MCMC for the LSU, SSU, TEF1-α, and RPB2 datasets, respectively, exhibited—although similar in their overall topology—various differences. However, none of the different relationships revealed by the separate analyses received reciprocal posterior probabilities (PP) of 99%, and, therefore, combining these four datasets was not considered to have any detrimental effect in estimating phylogenetic relationships among these taxa [60,68]. The *Serusiauxiella*, *Phylloporis*, *Puiggariella*, and *Raciborskiella* clades were monophyletic and strongly supported in all instances (no RPB2 data for the last). Based on nuSSU evidence, *Racoplaca* was nested within *Strigula prasina* with a posterior probability of 86%, whereas it was monophyletic with the LSU, TEF1-α, RPB2, and in the combined analysis (all 100% posterior probability). Considering that our selection of 99% as the threshold to determine if datasets should be combined, this has no detrimental effect. *Strigula* s.str. clade was recovered as monophyletic in the RPB2 and the concatenated dataset, but paraphyletic in the nuLSU and polyphyletic in nuSSU due to the invasion of *Racoplaca*. In the concatenated dataset, the six clades were resolved as monophyletic and with strong support, so we use this to reflect the most likely natural evolutionary relationship (Figure 2).

The four-marker tree, based on a concatenated alignment with 4373 bp (nuSSU: 1176 bp; nuLSU: 1274 bp; TEF1-α: 886 bp; RPB2: 1037 bp) also recovered the six genera with strong support (ML tree shown), and likewise recovered the genera *Puiggariella* and *Racoplaca*, and *Raciborskiella* and *Serusiauxiella* as sister clades, respectively (Figure 2). The clade including *Puiggariella* and *Racoplaca* had moderate support in the ITS (Figure 1) and no support in the four-marker tree (Figure 2), whereas the clade containing *Raciborskiella* and *Serusiauxiella* had no support in the ITS (Figure 1), but strong support in the four-marker tree (Figure 2). Stem branches were rather long for *Phylloporis*, *Raciborskiella*, and *Serusiauxiella*, moderately long for *Puiggariella* and *Racoplaca*, and comparatively short for *Strigula* s.str. (Figure 1 and Figure 2), agreeing with earlier findings [47]. Within the latter, both the ITS and the four-marker tree resolved the *S. nitidula* group on a long, strongly supported branch (Figure 1 and Figure 2).

The four-marker tree included data for one of the two new species in *Racoplaca* (*R. maculatoides*), and also resolved all nine new species in *Strigula* s.str. separately delimited based on the ITS tree (*S. guangdongensis*, *S. intermedia*, *S. laevis*, *S. microcarpa*, *S. pseudoantillarum*, *S. pseudosubtilissima*, *S. pycnoradians*, *S. sinoconcreta*, and *S. subtilissimoides*).

### 3.2. Species Tree Assessment

Phylogenetic analysis using concatenated datasets does not take into account the stochasticity of the coalescent process, and thus may fail to recover the true species tree [69]. Therefore, we also used a coalescent-based method to infer the species tree from a set of gene trees by explicitly taking into account the inherent stochasticity associated with the coalescent process [66]. 

**Figure 3:**
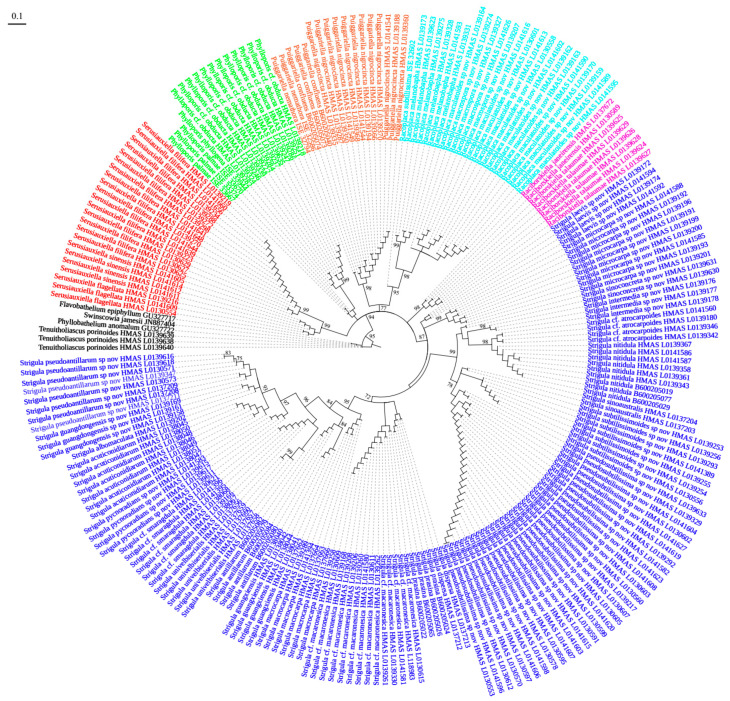
The coalescent-based species tree by ASTRAL.

The resulting ASTRAL tree (Figure 3) was quite similar to the single-marker ITS tree (Figure 1), except for the branch length patterns characteristic of the coalescent approach, supporting the usefulness of the ITS to delimit species in this clade and the congruence between the various markers. The genera also received good to strong support in the ASTRAL tree (87–99%), with the exception of *Phylloporis* (Figure 3). We further compared the interspecific topologies of each gene tree to the coalescent species tree topology and found that three of the five individual gene trees had partially different topologies, whereas the other two were congruent with the coalescent species tree topology.

### 3.3. Taxonomy

**Figure 4:**
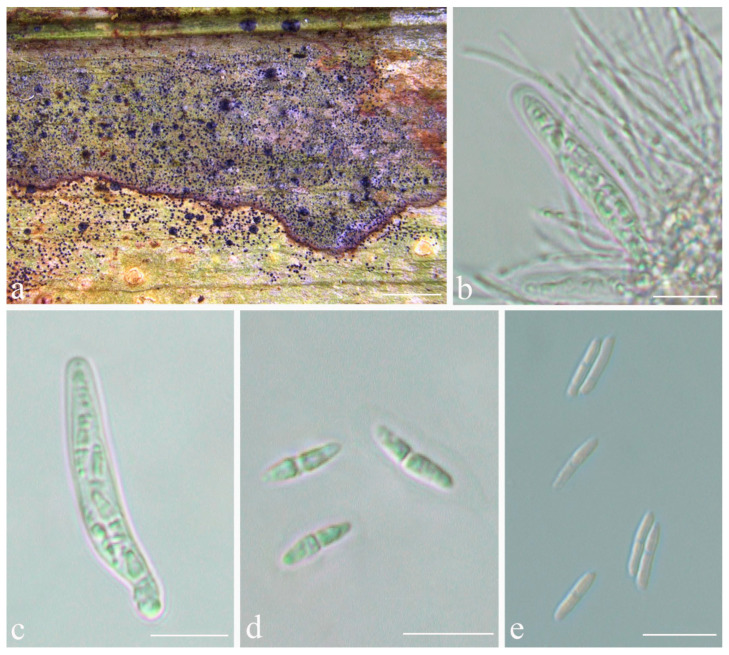
*Phylloporis palmae* (epitype, ISE–33670). (**a**) Thallus; (**b**), (**c**) asci; (**d**) ascospores; (**e**) macroconidia. Scale bars: (**a**) = 2 mm; (**b**–**e**) = 10 μm.

***Phylloporis palmae*** (Cavalc. & A.A. Silva) S.H. Jiang, J.C. Wei, Xavier-Leite & Lücking, **comb. nov.** (Figure 4).

*Basionym*: *Manaustrum palmae* Cavalc. & A.A. Silva, in Cavalcante et al., Publicações. Instituto de Micologia da Universidade de Pernambuco 647: 14. 1972.

MycoBank MB 838157.

*Typus*: BRAZIL, Amazonas, Manaus, Rodovia Manaus-Itacoatiara, 67 km; May 1961, Garnier s.n. (URM 23296/Exs. 15761, holotype!; INPA, isotype, not seen). Amapá, Floresta Nacional do Amapá, Municipio de Porto Grande, by bus 112 km from Macapá (145 NNW from Macapá), and then by boat 57 km from Porto Grande (70 km WNW from Porto Grande), S 01°02′32″, W 51°56′32″, Amazon rainforest, on leaves, August 2015, A.B. Xavier-Leite 2670 (ISE–33670, epitype, here designated; ITS Genbank No. MW344138; MBT 395040).

*Description*: Thallus supracuticular, continuous or marginally dispersed, 1–10 mm across and 10–15 μm thick, with entire margins, olive-green with metallic glance, densely furnished with minute, blackish verrucae. Photobiont *Phycopeltis* sp., cells rectangular, forming net-like plates with interspaces, 5–10 × 4–6 μm. Perithecia erumpent, hemispherical to wart-shaped, 0.2–0.3 mm diameter and 50–70 μm high, black or greyish black due to a thin thallus cover lacking algae. Excipulum prosoplectenchymatous, 7.5–10 μm thick, colourless to brownish. Involucrellum carbonaceous, 15–35 μm thick. Paraphyses unbranched, c. 1–1.5 μm thick. Asci cylindrical to narrowly obclavate, 28–45 × 5–7.5 μm. Ascospores eight per ascus, biseriate, fusiform-ellipsoid, one-septate, with slight constriction at septum, 8.5–10 × 2–3 μm, 3.5–4.5 times as long as broad. Pycnidia producing macroconidia present in the type material but not observed in the sequenced specimens, greyish black, wart-shaped, 0.1–0.15 mm in diameter. Macroconidia one-septate, 8–12 × 1.5–2 μm. Microconidia not seen.

*Chemistry*: No substances detected by TLC.

*Additional specimens examined*: BRAZIL, Amapá, Floresta Nacional do Amapá, Municipio de Porto Grande, by bus 112 km from Macapá, and then by boat 57 km from Porto Grande, S 01°02′32″, W 51°56′32″, Amazon rainforest, on leaves, August 2015, A.B. Xavier-Leite 2374 (ISE–33374), 2667 (ISE–33667). 

*Notes*: The sequenced material corresponds morphologically to *Phylloporis multipunctata* (G. Merr. ex R. Sant.) Vězda, described from Indonesia and recently synonymised under *P. cinefaciens* (Nyl.) S.H. Jiang, Lücking & Sérus. [41]. However, microscopic examination revealed that the foliicolous specimens from Brazil have much smaller ascospores than the type material of *P. cinefaciens* and *P. multipunctata* (12–16 × 3.5–4.5 µm in the latter two [41]). We, therefore, take up the epithet introduced by Cavalcante et al. [70], originally synonymised with *P. multipunctata* by Lücking (1998), for this material [71]. Since the holotype only bears pycnidia, we designate one of the sequenced specimens with numerous perithecia as epitype.

**Figure 5:**
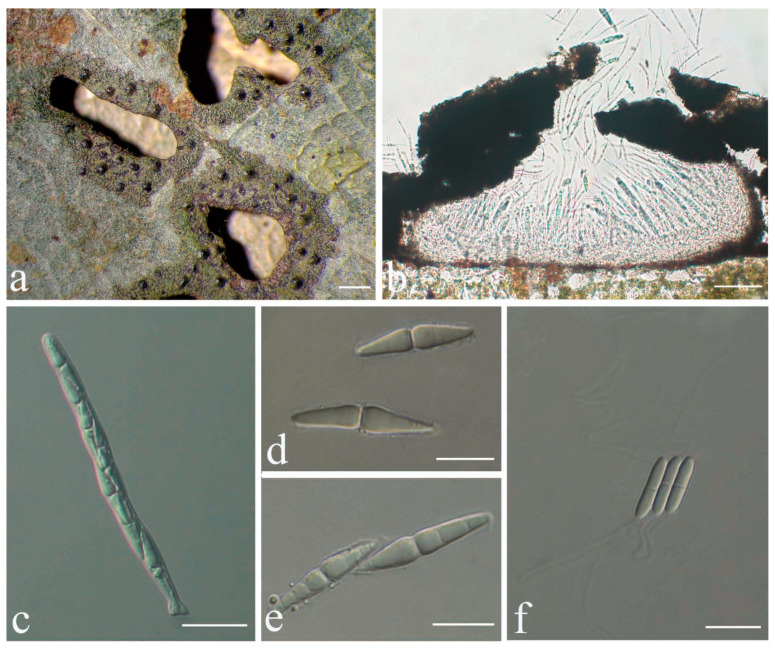
*Racoplaca macrospora* sp. nov. (holotype, HMAS–L0139274). (**a**) Thallus; (**b**) Perithecia; (**c**) asci; (**d**), (**e**) ascospores; (**f**) macroconidia. Scale bars: (**a**) = 1.5 mm; (**b**) = 50 μm; (**c**) = 20 μm; (**d**–**f**) = 10 μm.

***Racoplaca macrospora*** S.H. Jiang, J.C. Wei & Lücking, **sp. nov.** (Figure 5).

MycoBank MB 838159.

*Etymology*: The epithet macrospora refers to the large ascospores, thus far the largest known in the genus, strongly contrasting with the typically small thalli. 

*Typus*: CHINA, Guangxi, Shangsi County, Shiwan Mountain National Nature Reserve, N 21°53′50″, E 107°54′18″, alt. 411 m, on living leaves, 25 May 2015, X.L. Wei & J.H. Wang GX20150306 (HMAS–L0139274, holotype; ITS Genbank No. MW344166). 

*Diagnosis*: Differing from *Racoplaca maculata* (Cooke & Massee) S.H. Jiang, Lücking & J.C. Wei in the much larger, fusiform ascospores and the much larger, one-septate macroconidia. 

*Description*: Thallus subcuticular, continuous, growing along the margins and scars of leaves, lobes small, not very long, forming irregular lines, margin crenulate-laciniate, bordered by a thin, black line, dark (olive-)green, nitidous, 5–10 mm across and 10–20 μm thick. Photobiont a species of *Cephaleuros*, cells angular-rounded, 8–13 × 4–7 μm. Perithecia covered by thin thallus layer up to ostiole, wart-shaped, 0.2–0.45 mm diameter and 100–200 μm high, greyish to greenish black. Excipulum prosoplectenchymatous, 10–15 μm thick, colourless to brown. Involucrellum carbonaceous, 15–30 μm thick, black. Paraphyses unbranched, thin, about 1 μm. Asci narrowly obclavate, 75–100 × 7.5–10 μm, eight-spored. Ascospores biseriate or irregularly orientated, fusiform, one-septate, with distinct constriction at septum, 22.5–27.5 × 4–5 μm. Pycnidia exposed, wart-shaped, black and shiny, those producing macroconidia 0.1–0.2 mm, those producing microconidia 0.07–0.15 mm diameter. Macroconidia bacillar, one-septate, 10–15 × 1.8–2.5 μm, with 17–35 μm long appendage at both ends. Pycnidia producing microconidia appearing as tiny black points, or included in pycnidia producing macroconidia. Microconidia ellipsoid, 3–5 × 1 μm. 

*Chemistry*: No substances detected by TLC. 

*Habitat and distribution*: The new species was found in humid, semi-exposed forest habitats in southern China.

*Additional specimens examined*: CHINA, Guangxi, Shangsi County, Shiwan Mountain National Nature Reserve, N 21°53′50″, E 107°54′18″, alt. 411 m, on living leaves, 25 May 2015, X.L. Wei & J.H. Wang GX20150316 (HMAS–L0139327); Hainan, Ledong County, Jianfengling, Mingfenggu, N 18°44′5′′, E 108°52′8′′, alt. 920 m, on living leaves, 11 December 2014, J.H. Wang & R.D. Liu HN2014144_3 (HMAS–L0141626); 28 July 2009, J.C. Wei WJC065 (HMAS–L0139206), WJC069 (HMAS–L0139207).

*Notes*: In terms of thallus morphology, *Racoplaca macrospora* resembles *R. maculata* in the relatively compact, more greenish thallus lacking distinct laciniae. The latter, however, has much smaller (12–18 × 2.5–3.5 μm), oblong ascospores and much smaller, non-septate macroconidia [45]. The ascospores and macroconidia point to a closer relationship with *R. melanobapha* (Kremp.) S.H. Jiang, Lücking & J.C. Wei; however, that species has still smaller ascospores (14–22 × 3–5 μm), and also differs in the strongly and regularly laciniate, more brownish thallus [45]. This closer relationship is supported by the molecular data (Figure 1).

Based on the ITS data, *Racoplaca macrospora* was found nested within another newly recognised species, *R. maculatoides* (see below). However, there are between 18 consistent base call differences between the two species (substitutions and indels), corresponding to a mean identity value of 96% (Table S2), substantially below what could be accepted for within-species variation. Given that *R. maculatoides* has distinctly shorter (15–25 μm vs. 22.5–27.5 μm long) ascospores and macroconidia with shorter appendages (10–18 μm vs. 17–35 μm), we consider *R. macrospora* a recently emerging, yet distinct species. Species evolving from paraphyletic residuals are now broadly accepted [36], but an artifactually paraphyletic topology could also result from the most closely related species not having been sequenced yet. Another possible explanation is that *R. maculatoides* itself represents a species complex. Indeed, separate analysis of the ITS only for *Racoplaca* revealed three subclades in this clade: one supported subclade (75%), including the type of *R. maculatoides*, with a basally emerging individual with 63% support; another shallow subclade with four individuals currently assigned to *R. maculatoides*; and a strongly supported subclade (100%) on a long branch, representing *R. macrospora* (Figure S1).

**Figure 6:**
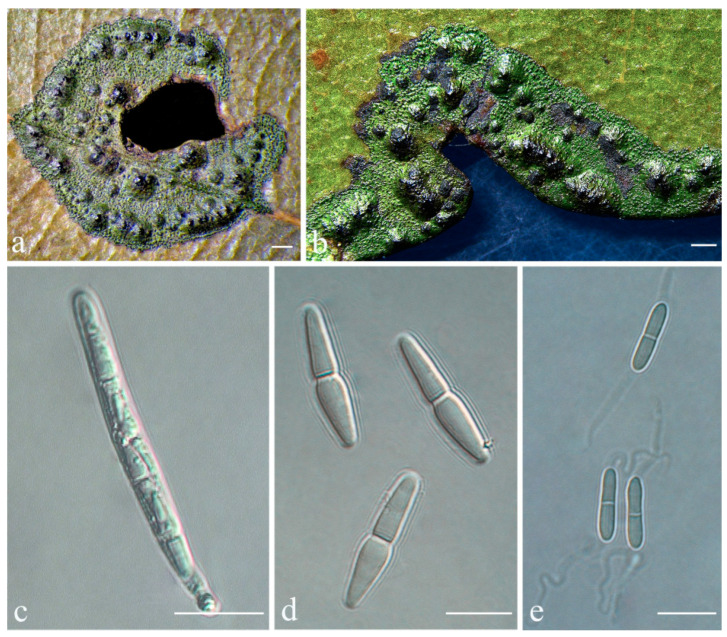
*Racoplaca maculatoides* sp. nov. (**a**), (**b**) Thallus (holotype, HMAS–L0139170); (**c**) ascus (holotype, HMAS–L0139170); (**d**) ascospores (holotype, HMAS–L0139170); (**e**) macroconidia (HMAS–L0139163). Scale bars: (**a**,**b**) = 500 μm, (**c**) = 20 μm, (**d**,**e**) = 10 μm.

***Racoplaca maculatoides*** S.H. Jiang, J.C. Wei & Lücking, **sp. nov.** (Figure 6).

MycoBank MB 838161.

*Etymology*: The epithet maculatoides refers to the morphological similarity with *Racoplaca maculata*.

*Typus*: CHINA, Guangdong, Shixing County, Chebaling National Nature Reserve, N 24°43′27″, E 114°15′22″, alt. 345 m, on living leaves, 14 May 2015, X.L. Wei & J.H. Wang GD2015020 (HMAS–L0139170, holotype; ITS Genbank No. MW344174). 

*Diagnosis*: The new species externally resembles *Racoplaca maculata* but differs by the longer asci, larger, fusiform ascospores, and larger, one-septate macroconidia. 

*Description*: Thallus subcuticular, continuous, sometimes occurring along the nerves, margins, and scars of leaves, lobes confluent, indicated by irregular, dark lines and dots on the thallus surface, margin entire or slightly crenulate, bordered by thin, black line, dark green, nitidous, with a metallic glance, 5–15 mm across and 10–20 μm thick. Photobiont a species of *Cephaleuros*, cells angular-rounded, 8–13 × 4–7 μm. Perithecia covered by thin thallus layer up to ostiole, wart-shaped, 0.25–0.5 mm diameter and 100–200 μm high, greyish to greenish black. Excipulum prosoplectenchymatous, 10–15 μm thick, colourless to brown. Involucrellum carbonaceous, 15–30 μm thick, black. Paraphyses unbranched or slightly branched, thin. Asci narrowly obclavate, 50–90 × 7.5–10 μm, eight-spored. Ascospores biseriate or irregularly orientated, fusiform, one-septate, with slight constriction at the septum, 15–25 × 3–5 μm. Pycnidia exposed, wart-shaped, black and shiny, those producing macroconidia 0.1–0.2 mm diameter, those producing microconidia 0.07–0.15 mm diameter. Macroconidia bacillar, one-septate, 10–12.5 × 2.5 μm, with 10–18 μm long appendage at both ends that may be terminated by a hook. Pycnidia producing microconidia appearing as tiny black points, or included in pycnidia producing macroconidia. Microconidia ellipsoid, 3–5 × 1 μm. 

*Chemistry*: No substances detected by TLC. 

*Habitat and distribution*: The new species was found in humid, semi-exposed forest habitats in southern China. 

*Additional specimens examined*: CHINA, Guangdong, Shixing County, Chebaling National Nature Reserve, N 24°43′27″, E 114°15′22″, alt. 345 m, on living leaves, 14 May 2015, X.L. Wei & J.H. Wang GD2015025_2 (HMAS–L0139159), GD2015025_8 (HMAS–L0139162), GD2015025_9 (HMAS–L0139163), GD2015025_10 (HMAS–L0139164); N 24°42′35″, E 114°13′37″, alt. 398 m, on living leaves, 14 May 2015, X.L. Wei & J.H. Wang GD2015031_7_1 (HMAS–L0141589); N 24°42′39″, E 114°13′30″, alt. 387 m, on living leaves, 15 May 2015, X.L. Wei & J.H. Wang GD2015038_2_1 (HMAS–L0141590); Hainan, Ledong County, Jianfengling, Mingfenggu, N 18°44′5″, E 108°52′8″, alt. 920 m, on living leaves, 11 December 2014, J.H. Wang & R.D. Liu HN2014123 (HMAS–L0130586), HN2014123_5 (HMAS–L0141595), HN2014144 (HMAS–L0130598), HN2014177 (HMAS–L0130558), HN2014134 (HMAS–L0130591), HN2014148 (HMAS–L0130601), HN2014136 (HMAS–L0130592); N 18°44′36″, E 108°50′39″, alt. 962 m, on living leaves, 12 December 2014, J.H. Wang & R.D. Liu HN2014221 (HMAS–L0130606), HN2014221_4 (HMAS–L0141602); N 18°44′32″, E 108°50′32″, alt. 985 m, on living leaves, 12 December 2014, J.H. Wang & R.D. Liu HN2014286_2 (HMAS–L0141616), HN2014281_1 (HMAS–L0141613); Qiongzhong County, Limu mountain, N 19°10′29″, E 109°44′53″, alt. 800 m, on living leaves, 10 September 2017, S.H. Jiang HN20170656 (HMAS–L0139637).

*Notes*: *Racoplaca maculatoides* strongly resembles *R. maculata* in morphology but can be distinguished by the longer asci; larger, fusiform ascospores; and larger, one-septate macroconidia (asci 40–60 × 5–8 μm, ascospores 12–18 × 2.5–3.5 μm, macroconidia aseptate, 4–6 × 1.5–2 μm in *R. maculata* [45]). *Racoplaca maculatoides* is most similar and most closely related to *R. macrospora* (Figure 1). It differs by the shorter ascospores and the narrower macroconidia with shorter appendages. *Racoplaca melanobapha* resembles *R. maculatoides* in ascospores and macroconidia, but differs in the strongly and regularly laciniate, more brownish thallus [45]. Both are also phylogenetically distinct (Figure 1 and Figure 2).

*Racoplaca melanobapha* (Kremp.) S.H. Jiang, Lücking & J.C. Wei, comb. nov.

MycoBank MB 842243.

*Basionym*: *Verrucaria melanobapha* Kremp., Lichenes Foliicolae Quos legit O. Beccari Annis 1866–1867 in Insula Borneo: 18 (1874); *Verrucaria melanobapha* Kremp., Nuovo Giornale Botanico Italiano 7: 51 (1875) (nom. inval., ICN Art. 6.3 Note 2); *Strigula melanobapha* (Kremp.) R. Sant., Symb. Bot. Upsal. 12(1): 188 (1952).

*Typus*: Malaysia (Borneo), *Beccari 219* (M, holotype!).

*Notes*: This combination was first proposed by Jiang et al. [47] but was unfortunately invalid as the basionym was not cited. It is, therefore, validated here.

**Figure 7:**
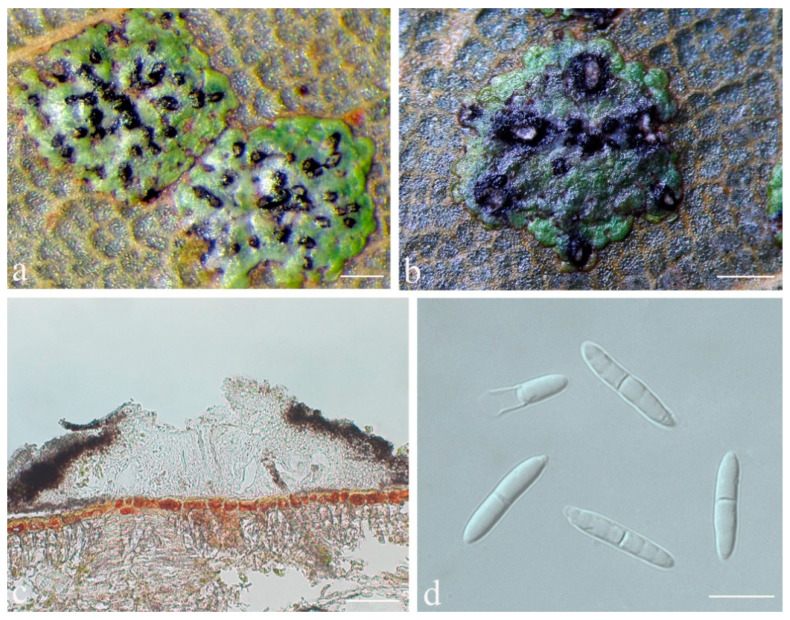
*Strigula guangdongensis* sp. nov. (holotype, HMAS–L0139169). (**a**,**b**) Thallus; (**c**) perithecia; (**d**) macroconidia. Scale bars: (**a**,**b**) = 200 μm, (**c**) = 50 μm, (**d**) = 10 μm.

***Strigula guangdongensis*** S.H. Jiang, J.C. Wei & Lücking, **sp. nov.** (Figure 7).

MycoBank MB 838162.

*Etymology*: The specific epithet is derived from the name of the type locality of the species, Guangdong province.

*Typus*: CHINA, Guangdong, Shixing County, Chebaling National Nature Reserve, N 24°43′27″, E 114°15′22″, alt. 345 m, on living leaves, 14 May 2015, X.L. Wei & J.H. Wang GD2015019 (HMAS–L0139169, holotype; ITS Genbank No. MW344202). 

*Diagnosis*: The new species can be distinguished by the beaked, more elongate pycnidia than in other species of the genus. The phylogenetic trees also indicate it forms an independent clade. 

*Description*: Thallus subcuticular, 0.5–4 mm across and 15–25 μm thick, continuous or dispersed into rounded to irregular, partly confluent patches, with entire to crenulate or lobulate margins, bright green mottled with white. Photobiont a species of *Cephaleuros*, cells angular-rounded, 5–15 × 4–10 μm. Perithecia few, 0.1–0.3 mm diameter and 120–180 μm high, postmature; no asci or ascospores observed. Pycnidia numerous, conical to shortly beaked, 40–60 μm diameter, black, the beak paler and somewhat translucent. Macroconidia bacillar, one-septate, sometimes breaking into halves, 12.5–20 × 3–4 μm. Microconidia not seen. 

*Chemistry*: No substances detected by TLC.

*Habitat and distribution*: It was found on the surface of living leaves in humid, semi-exposed forests of south China. 

*Additional specimens examined*: CHINA, Guangdong, Shixing County, Chebaling National Nature Reserve, N 24°43′27″, E 114°15′22″, alt. 345 m, on living leaves, 14 May 2015, X.L. Wei & J.H. Wang GD2015025_7 (HMAS–L0139161), GD2015025_1 (HMAS–L0139158).

*Notes*: This is another newly recognised species that belongs in the morphologically defined *Strigula smaragdula* complex. The latter has been described from Nepal and we have currently marked one phylogenetically distinct clade from China as a candidate for this species (*S.* cf. *smaragdula*; Figure 1 and Figure 2). *Strigula guangdongensis* differs from this clade phylogenetically and is also set apart by its beaked pycnidia. Lücking also mentioned specimens of *S. smaragdula* s.lat. with shortly beaked pycnidia from the Neotropics, but these likely represent another unrecognised taxon [45]. A corticolous species from Madagascar, *S. rostrata* R.C. Harris & Aptroot, has more strongly beaked pycnidia and submuriform macroconidia [43]; that species is now placed in a different genus, as *Swinscowia rostrata* (R.C. Harris & Aptroot) S.H. Jiang, Lücking & Sérus. [41].

**Figure 8:**
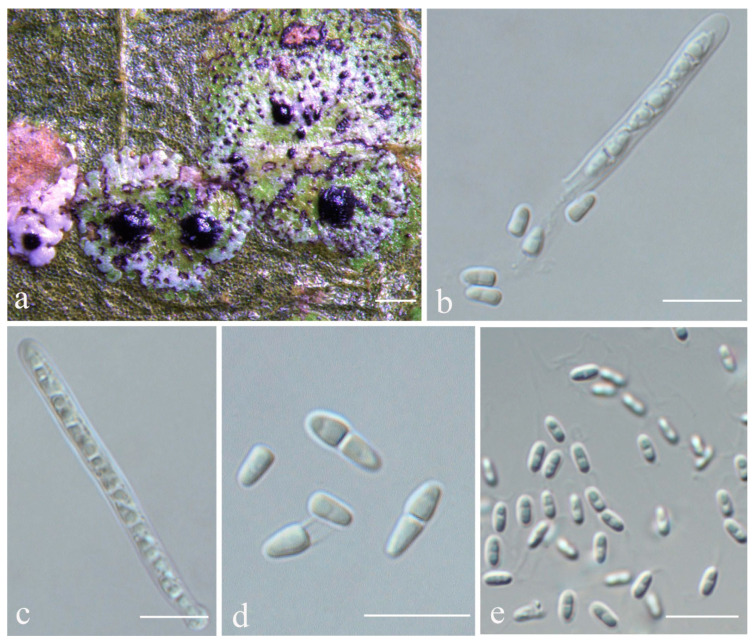
*Strigula intermedia* sp. nov. (holotype, HMAS–L0139177). (**a**) Thallus; (**b**,**c**) asci and ascospores; (**d**) ascospores; (**e**) macroconidia. Scale bars: (**a**) = 300 μm, (**b**–**e**) = 10 μm.

***Strigula intermedia*** S.H. Jiang, J.C. Wei & Lücking, **sp. nov.** (Figure 8).

MycoBank MB 838163.

*Etymology*: The epithet refers to the thallus being intermediate between that of *Strigula concreta* and *S. nitidula*.

*Typus*: CHINA, Yunnan, Pu’er city, Simao District S214, alt. 829 m, on living leaves, 23 October 2016, X.Y. Liu YN20160161 (HMAS–L0139177, holotype; ITS Genbank No. MK206305). 

*Diagnosis*: The new species is intermediate between *Strigula concreta* (Fée) R. Sant. s.str. and *S. nitidula* Mont. s.str. in having a slightly thickened thallus, with irregular, short black lines.

*Description*: Thallus subcuticular, 1.5–4.5 mm across and 7.5–22.5 μm thick, with entire to crenulate margins, bright green mottled with white, surface smooth, sometimes with black dots or lines. Photobiont a species of *Cephaleuros*, cells angular-rounded, 8–12 × 4–6 μm. Perithecia exposed, conical to wart-shaped, 0.25–0.5 mm in diameter and 100–190 μm high, black. Excipulum prosoplectenchymatous, 12.5–25 μm thick, brown. Involucrellum 25–62.5 μm thick, carbonaceous, black. Paraphyses unbranched. Asci cylindrical, 40–60 × 3–4 μm. Ascospores eight per ascus, uniseriate, ellipsoid, one-septate, sometimes with one to two oil droplets per cell when fresh, with distinct constriction at the septum and sometimes broken into parts outside the asci, 7.5–10 × 2–2.5 μm. Pycnidia wart-shaped, those producing macroconidia 0.1–0.15 mm, those producing microconidia 0.05–0.1 mm diameter, black. Macroconidia bacillar, zero- to one-septate, 4–6 × 1.5–2.5 μm, with appendage at one end or both ends c. 5–10 μm long. Microconidia fusoid, aseptate, 4–5 × 1.5–2 μm.

*Chemistry*: No substances detected by TLC. 

*Habitat and distribution*: The new species grows on living leaves in wet tropical forest in China. 

*Additional specimens examined*: CHINA, Yunnan, Pu’er city, Simao District S214, alt. 829 m, on living leaves, 23 October 2016, X.Y. Liu YN20160160 (HMAS–L0139176), YN20160162 (HMAS–L0139178). 

*Notes*: *Strigula intermedia* conforms to the morphology of *S. concreta*, in particular the rather thin thallus with crenulate margins, the exposed, black perithecia, and the uniseriate asci with short ascospores sometimes breaking into halves [45]. There is at least one other new species belonging to this morphodeme, *S. sinoconcreta* (see below). Given that both are phylogenetically distinct in both the ITS and the four-marker phylogeny (Figure 1 and Figure 2), we have to assume that *S. concreta* s.lat. forms a species complex, similar to *S. smaragdula* and intermingled with lineages representing the *S. nitidula* morphodeme (see below). The latter agrees with *S. concreta* anatomically, including the uniseriate asci with short ascospores often breaking into halves, but has a very thin thallus often bordered by a black line, as in *Racoplaca* [42,45]. The two thallus morphologies are sometimes difficult to distinguish, a feature attributed to leaf characteristics [45], but it appears that numerous lineages are involved. *Strigula intermedia* represents an intermediate thallus type, thinner than in typical *S. concreta* but thicker than in typical *S. nitidula*, and with short, irregular black lines not as distinct as in *S. nitidula*.

*Strigula concreta* has been described from the Caribbean, with one current synonym from Brazil (*S. rugulosa* Müll. Arg.), one from Africa (*S. atrocarpa* Vain.), and two from the Philippines (*S. sulcata* Vain., *Porina crenulata* Vain. [42,45]). These names are, therefore, potentially available for cryptic or near-cryptic lineages in the corresponding regions. However, considering the striking diversification of the *S. smaragdula* complex in Asia alone, with no apparent overlap in species distributions between (sub)tropical China and Korea or Thailand ([48,49,50], this paper), it seems unlikely that the Philippine material of *Strigula sulcata* or *Porina crenulata* is conspecific with any of the two Chinese lineages detected here, and, therefore, we introduce new species for the latter.

**Figure 9:**
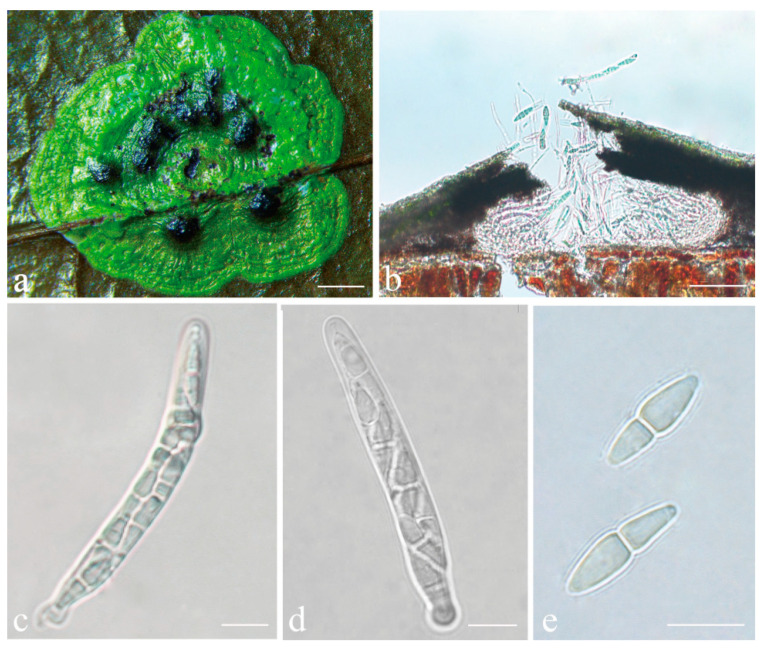
*Strigula laevis* sp. nov. (holotype, HMAS–L0141592). (**a**) Thallus; (**b**) perithecia; (**c**,**d**) asci; (**e**) ascospores. Scale bars: (**a**) = 500 μm, (**b**) = 50 μm, (**c**) = 20 μm, (**d**,**e**) = 10 μm.

***Strigula laevis*** S.H. Jiang, J.C. Wei & Lücking, **sp. nov.** (Figure 9).

MycoBank MB 838164.

*Etymology*: Epithet of the new species “laevis” is a Latin word, and means smooth and bright thallus.

*Typus*: CHINA, Guangdong, Ruyuan county, Nanling National Forest Park, N 24°54′59″, E 113°2′5″, alt. 848 m, on living leaves, 17 May 2015, X.L. Wei & J.H. Wang GD2015066_2 (HMAS–L0141592, holotype; ITS Genbank No. MW344203). 

*Diagnosis*: The species is morphologically similar to *Strigula nigrocarpa* Lücking, but can be distinguished by the small, thin thallus, not sharply delimited perithecia, and biseriate, smaller ascospores.

*Description*: Thallus subcuticular, continuous, 2–5 mm across and 10–20 μm thick, with entire margins, bright to dark green, smooth. Photobiont a species of *Cephaleuros*, cells angular-rounded, 6–15 × 6–10 μm. Perithecia covered by a thin thallus layer, applanately hemispherical, 0.3–0.5 mm diameter and 100–180 μm high, dark green but uppermost part exposed and black. Excipulum prosoplectenchymatous, 5–10 μm thick, colourless to brown. Involucrellum carbonaceous, 12.5–32.5 μm thick, black. Paraphyses unbranched. Asci obclavate, 52–80 × 6–10 μm, eight-spored. Ascospores biseriate, oblong-ellipsoid, one-septate, with slight constriction at septum, 16–20 × 3–5 μm. Pycnidia not seen. 

*Chemistry*: No substances detected by TLC. 

*Habitat and distribution*: This species was found on living leaves in tropical to subtropical rainforests of southern China. 

*Additional specimens examined*: CHINA, Guangdong, Ruyuan county, Nanling National Forest Park, N 24°54′59″, E 113°2′5″, alt. 848 m, on living leaves, 17 May 2015, X.L. Wei & J.H. Wang GD2015066 (HMAS–L0139172), GD2015066_4 (HMAS–L0141594). Hunan, Mangshan National Forest Park, N 24°56′59″, E 112°56′2″, alt. 1257 m, on living leaves, 17 May 2015, X.L. Wei & J.H. Wang GD2015077 (HMAS–L0139174). 

*Notes*: *Strigula laevis* belongs in the *S. smaragdula* complex and is somewhat similar to the neotropical *S. nigrocarpa*, due to the blackish perithecia. However, in *S. nigrocarpa*, the perithecia are sharply delimited from the thallus, and the ascospores are uniseriate in longer, cylindrical asci, and the ascospores are somewhat larger [45]. It is also similar to the neotropical *S. minuta* Lücking, but that species has much smaller ascospores (9–12 × 2–3 μm [45]).

**Figure 10:**
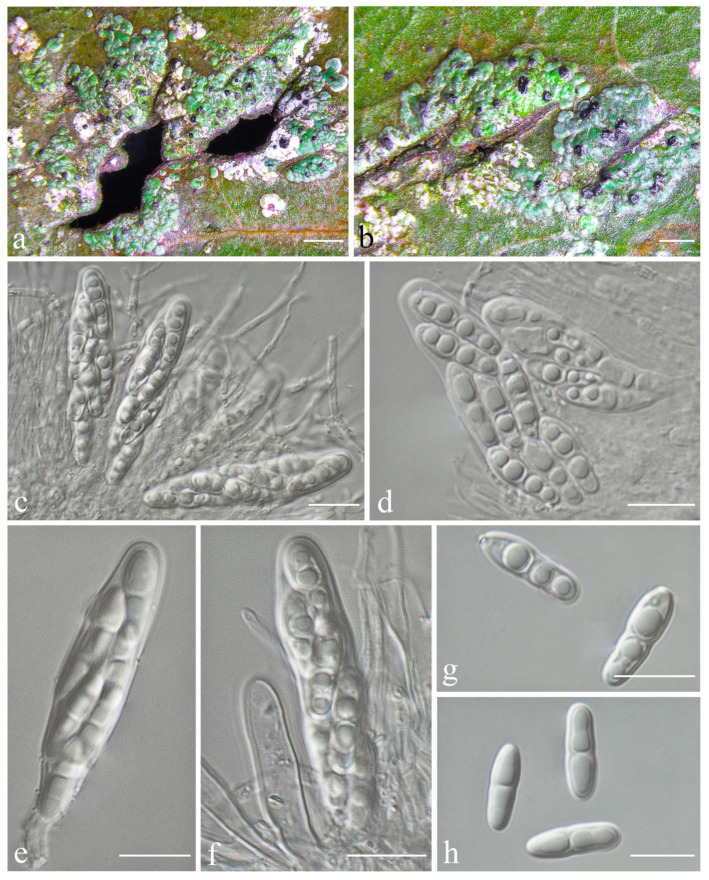
*Strigula microcarpa* sp. nov. (holotype, HMAS–L0139196). (**a**,**b**) Thallus; (**c**–**f**) asci and ascospores; (**g**,**h**) ascospores. Scale bars: (**a**) = 1000 μm, (**b**) = 500 μm, (**c**–**h**) = 10 μm.

***Strigula microcarpa*** S.H. Jiang, J.C. Wei & Lücking, **sp. nov.** (Figure 10).

MycoBank MB 838165.

*Etymology*: Epithet of the new species “microcarpa” is a Latin word, and means small perithecia.

*Typus*: CHINA, Yunnan, Xishuangbanna, Jinghong city, N 22°19′15″, E 100°47′13″, alt. 968 m, on living leaves, 28 October 2016, X.Y. Liu YN20160099 (HMAS–L0139196, holotype; ITS Genbank No. MW344208). 

*Diagnosis*: The new species was similar with *Strigula microspora* Lücking in the thallus, but can be distinguished by the shorter ascus and the biseriate ascospores. 

*Description*: Thallus subcuticular, continuous, 3–7 mm across and 20–30 μm thick, typically with crenulate or shortly lobulate margins, pale greyish green to whitish. Photobiont a species of *Cephaleuros*, cells angular-rounded, 5–17 × 4–11 μm. Perithecia immersed-erumpent, hemispherical, 0.2–0.3 mm diameter and 100–180 μm high, exposed part black. Excipulum prosoplectenchymatous, 5–10 μm thick, blackish brown. Involucrellum confluent with excipulum, carbonaceous, 20–40 μm thick, black. Paraphyses unbranched or slightly branched, thin. Asci cylindrical, 35–55 × 8.5–10 μm. Ascospores eight per ascus, biseriate to irregularly arranged, fusiform to ellipsoid, one-septate, with slight constriction at septum, 12.5–15 × 3.5–4.5 μm, 3–5 times as long as broad. Pycnidia not seen. 

*Chemistry*: No substances detected by TLC. 

*Habitat and distribution*: Collected on living leaves in humid, semi-exposed forests of southern China. 

*Additional specimens examined*: CHINA, Yunnan, Mengla county, N 21°42′10″, E 101°37′28″, alt. 669 m, on living leaves, 24 October 2016, X.Y. Liu YN20160154_2 (HMAS–L0141585); Pu’er city, Hani and Yi Autonomous County of Jiangcheng, N 22°38′56″, E 101°24′27″, alt. 829 m, on living leaves, 23 October 2016, X.Y. Liu YN20160161_1 (HMAS–L0141588); Pu’er city, Simao district S214, alt. 1316 m, on living leaves, 23 October 2016, X.Y. Liu YN20160044 (HMAS–L0139175); Xishuangbanna, Jinghong city, N 22°19′15″, E 100°47′13″, alt. 968 m, on living leaves, 28 October 2016, X.Y. Liu YN20160097 (HMAS–L0139191), YN20160098 (HMAS–L0139192), YN20160102 (HMAS–L0139199), YN20160103 (HMAS–L0139193), YN20160105 (HMAS–L0139200), YN20160106 (HMAS–L0139201). 

*Notes*: *Strigula microcarpa* bears some resemblance to the neotropical species *S. microspora*, but the latter has longer asci (50–70 × 5–6 μm) and uniseriate ascospores [45]. *Strigula wandae* M. Cáceres & Lücking from the Valdivian Forest in Chile also resembles *S. microcarpa* in morphology, but has larger ascospores (15–23 × 4–5 μm [45]). While the ITS resolved the new species as an early diverging lineage within *Strigula* s.str. (Figure 1), the four-marker tree revealed a strongly supported relationship with the *S. prasina* Müll. Arg. complex (Figure 2).

**Figure 11:**
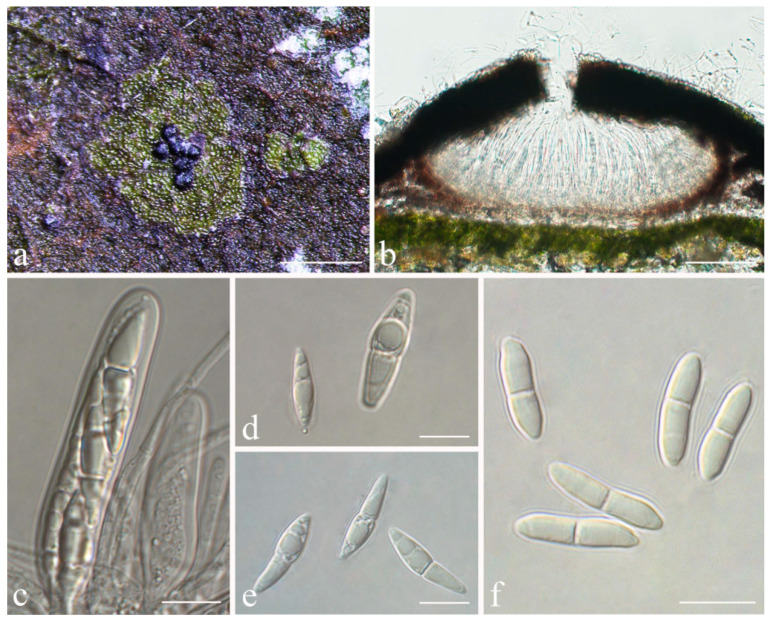
*Strigula pseudoantillarum* sp. nov. (**a**) Thallus with pycnidia (holotype, HMAS–L0137209); (**b**) perithecia (holotype, HMAS–L0137209); (**c**) ascus with biseriate ascospores (holotype, HMAS–L0137209); (**d**) ascospores (holotype, HMAS–L0137209); (**e**) ascospores (HMAS–L0137211); (**f**) macroconidia (HMAS–L0137211). Scale bars: (**a**) = 200 μm, (**b**) = 50 μm, (**c**–**f**) = 10 µm.

***Strigula pseudoantillarum*** S.H. Jiang, J.C. Wei & Lücking, **sp. nov.** (Figure 11).

MycoBank MB 838166.

*Etymology*: The epithet refers to the potential confusion with *Strigula antillarum*.

*Typus*: CHINA, Guangxi, Nanning, Long’an County, Longhu Mountain Natural Reserve, N 22°57′42″, E 107°37′40″, alt. 147 m, on living leaves, 1 December 2015, S.H. Jiang GX201511110 (HMAS–L0137209, holotype; ITS Genbank No. KX216696).

*Diagnosis*: *Strigula pseudoantillarum* is similar to *S. antillarum* (Fée) Müll. Arg. regarding the ascospores and aggregated pycnidia producing macroconidia, but it has a thin thallus and is also phylogenetically different from the latter. 

*Description*: Thallus subcuticular, medium green and with a somewhat metallic glance, 0.5–7.5 mm diameter and 12.5–27.5 μm thick, with entire to crenulate margins, green. Photobiont a species of *Cephaleuros*, cells angular-rounded, 6–15 × 6–10 μm. Perithecia covered by thallus, hemispherical, 0.3–0.6 mm diameter and 120–180 μm high. Excipulum prosoplectenchymatous, 5–12.5 μm thick, colourless to brown. Involucrellum carbonaceous, 10–63 μm thick, black. Paraphyses unbranched. Asci obclavate, 62–75 × 10–12.5 μm. Ascospores eight per ascus, biseriate, fusiform, with acute ends, 15–28 × 4–6 μm. Pycnidia producing macroconidia often aggregated and confluent in the centre of thallus patches, wart-shaped, 0.1–0.15 mm diameter, black. Macroconidia bacillar, one-septate, 15–20 × 3–4 μm. Pycnidia producing microconidia not seen. 

*Chemistry*: No substances detected by TLC.

*Distribution and ecology*: The new species was found on living leaves in humid, semi-exposed forest habitats of southern China.

*Additional specimens examined*: CHINA, Guangxi, Nanning, Long’an County, Longhu Mountain Natural Reserve, N 22°57′42″, E 107°37′40″, alt. 147 m, on living leaves, 1 December 2015, S.H. Jiang GX201511112 (HMAS–L0137208), GX201511114 (HMAS–L0137207), GX201511137 (HMAS–L0137667), GX201511166 (HMAS–L0137669), GX201511168 (HMAS–L0137668); Hainan, Dongfang city, Nanlang village, E’xian Ling, N 19°00′18″, E 109°04′09″, alt. 160 m, on living leaves, 13 December 2014, J.H. Wang & R.D. Liu HN2014376 (HMAS–L0130571), HN2014354 (HMAS–L0130622); N 19°00′07″, E 109°04′20″, alt. 126 m, J.H. Wang & R.D. Liu HN2014377 (HMAS–L0130572); Yunnan, Xishuangbanna, Mengla County, Tropical Botanical Garden of Chinese Academy of Sciences, East area, N 21°55′39″, E 101°15′52″, alt. 560 m, on living leaves, 18 November 2015, X.L. Wei & S.H. Jiang XTBG2015051 (HMAS–L0137211); Lvshilin, N 21°54′35″, E 101°16′52″, alt. 626 m, 18 November 2015, X.L. Wei & S.H. Jiang XTBG2015119 (HMAS–L0137210). 

*Notes*: *Strigula pseudoantillarum* is characterised by its aggregated, confluent pycnidia in the centre of the thallus. Together with the similar asci and ascospores, it thus resembles *S. antillarum*, and the material was originally reported under this name from China [48]. However, inclusion of authentic material of *S. antillarum* from the Caribbean, the type region, showed that both taxa are phylogenetically distinct and not even closely related (Figure 1 and Figure 2). A consistent morphological difference is found in the thin thallus in the new species, compared to the thicker, somewhat bulging thallus patches in typical *S. antillarum* [48]. *Strigula lacericola* P.M. McCarthy, described from Australia, can be distinguished by the pseudostromatic groups of pycnidia and by the smaller ascospores and macroconidia [72].

**Figure 12:**
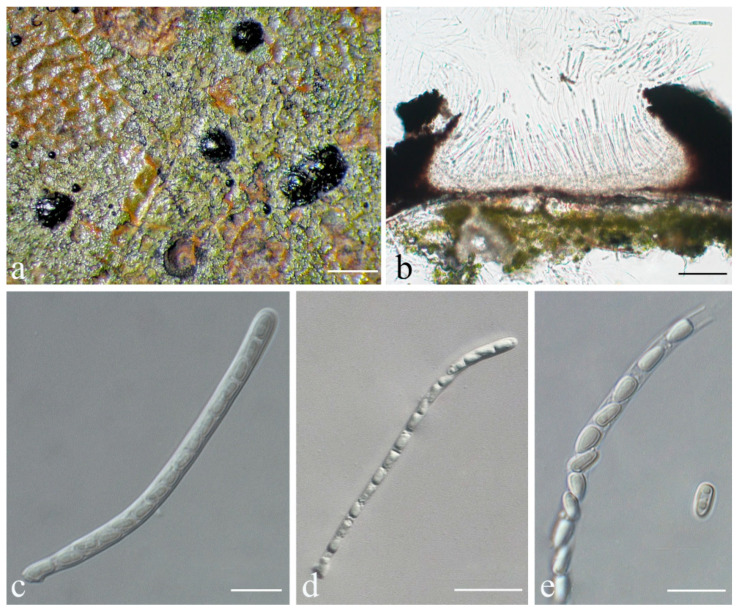
*Strigula pseudosubtilissima* sp. nov. (**a**) Thallus (holotype, HMAS–L0130553); (**b**) perithecia (holotype, HMAS–L0130553); (**c**) ascus (holotype, HMAS–L0130553); (**d**) ascus (HMAS–L0130597); (**e**) ascospores (holotype, HMAS–L0130553). Scale bars: (**a**) = 600 μm, (**b**) = 50 μm, (**c**,**e**) = 10 μm, (**d**) = 20 μm.

***Strigula pseudosubtilissima*** S.H. Jiang, J.C. Wei & Lücking, **sp. nov.** (Figure 12).

MycoBank MB 838167.

*Etymology*: The epithet pseudosubtilissima refers to the similarity with the unrelated *Racoplaca subtilissima*. 

*Typus*: CHINA, Hainan, Ledong County, Jianfengling, Mingfenggu, N 18°44′5″, E 108°52′8″, alt. 920 m, on living leaves, 11 December 2014, J.H. Wang & R.D. Liu HN2014130 (HMAS–L0130553, holotype; ITS Genbank No. MW344227). 

*Diagnosis*: *Strigula pseudosubtilissima* is similar to *Racoplaca subtilissima* Fée in the thin, laciniate thallus bordered by a black line, but can be distinguished by the uniseriate, short ascospores, as well as the green thallus colour and fully exposed, black perithecia. It differs from the closely related *S. nitidula* in the rather long, free lobes. 

*Description*: Thallus subcuticular, marginally with rather long, free laciniae, bordered by a thin, irregular, black line, 6–10 mm across and 7.5–12.5 μm thick, bright green with metallic glance. Photobiont a species of *Cephaleuros*, cells 8–14 × 4–6 μm. Perithecia completely exposed, almost conical, 0.2–0.6 mm diameter and 100–200 μm high, black and shiny. Excipulum prosoplectenchymatous, 10–20 μm thick, colourless to brown. Involucrellum carbonaceous, black, 15–30 μm thick. Paraphyses unbranched. Asci cylindrical or almost thread-like, very numerous and compact, 75–90 × 4–5 μm. Ascospores eight per ascus, uniseriate, ellipsoid, one-septate, with distinct constriction at septum and often broken into halves within the asci, 8–12 × 2.5–3 μm, 3–4 times as long as broad. Pycnidia usually rather few, often empty, 0.05–0.15 mm diameter. Pycnidia producing macroconidia and those producing microconidia are difficult to distinguish from their exterior. Macroconidia not seen. Microconidia fusiform, non-septate, 4–5 × 1.5–2 μm.

*Chemistry*: No substances detected by TLC.

*Habitat and distribution*: The new species was found on living leaves in humid, semi-exposed forest in southern China.

*Additional specimens examined*: CHINA, Guangxi, Shangsi County, Shiwan Mountain National Nature Reserve, N 21°54′13″, E 107°54′13″, alt. 264 m, on living leaves, 5 December 2015, S.H. Jiang GX201511356 (HMAS–L0139292); N 21°54′16″, E 107°54′09″, alt. 271 m, on living leaves, 26 May 2015, X.L. Wei & J.H. Wang GX20150341 (HMAS–L0139329), GX20150341_3 (HMAS–L0141582); Hainan, Ledong County, Jianfengling, Mingfenggu, N 18°44′49″, E 108°51′44″, alt. 828 m, on living leaves, 12 December 2014, J.H. Wang & R.D. Liu HN2014312_2 (HMAS–L0141619), HN2014256 (HMAS–L0130570), HN2014224 (HMAS–L0130607); N 18°44′5″, E 108°52′8″, alt. 920 m, on living leaves, 11 December 2014, J.H. Wang & R.D. Liu HN2014134 (HMAS–L0130591), HN2014141 (HMAS–L0130597); N 18°44′32″, E 108°50′32″, alt. 985 m, on living leaves, 12 December 2014, J.H. Wang & R.D. Liu HN2014284 (HMAS–L0130614); N 18°44′22″, E 108°51′58″, alt. 845 m, on living leaves, 11 December 2014, J.H. Wang & R.D. Liu HN2014009 (HMAS–L0130578); Lingshui, Diaoluo Mountain National Nature Reserve, N 18°43′36″, E 109°52′06″, alt. 930 m, on living leaves, 31 October 2016, X.Y. Liu HN2016003 (HMAS–L0139217); Qiongzhong County, Limu mountain, N 19°10′29″, E 109°44′53″, alt. 800 m, on living leaves, 10 September 2017, S.H. Jiang HN20170609 (HMAS–L0139633).

*Notes*: At first, this new species resembles *Racoplaca subtilissima*, but it differs in the green thallus, the fully exposed, black perithecia, and the uniseriate, short ascospores breaking into halves. These latter features point to a close relationship with *S. nitidula*, which is supported by the molecular data. *Strigula nitidula* s.lat. often forms marginal black lines, akin to *R. subtilissima*, but differs in the above features from the latter [45]. *Strigula nitidula* was described from the Caribbean (Cuba), and the ITS data suggest that Cuban and Chinese material form a homogeneous clade representing a single species (Figure 1), thus far the only species within the family demonstrated to be genuinely pantropical. Both the ITS data and the four-marker tree show that *S. pseudosubtilissima* is phylogenetically distinct (Figure 1 and Figure 2), also differing from typical *S. nitidula* morphologically in the rather long, partly free marginal laciniae.

**Figure 13:**
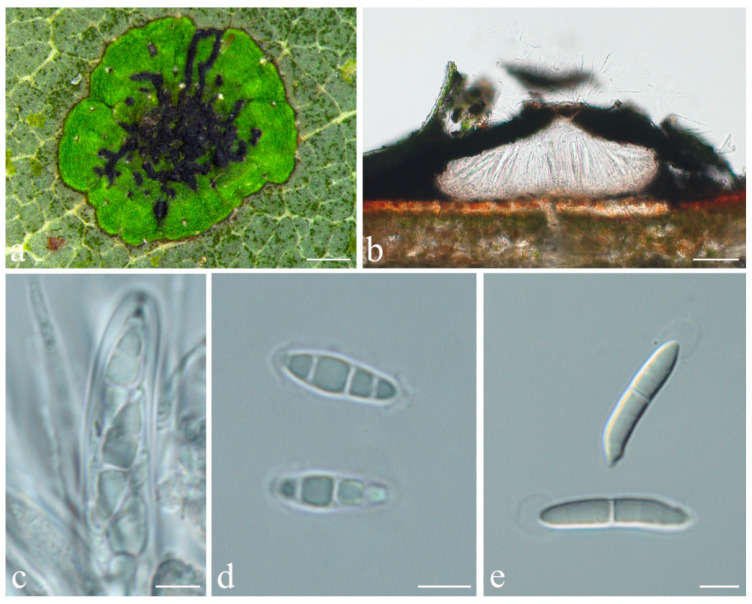
*Strigula pycnoradians* sp. nov. (holotype, HMAS–L0139611). (**a**) Thallus; (**b**) perithecia; (**c**) asci; (**d**) ascospores; (**e**) macroconidia. Scale bars: (**a**) = 300 μm, (**b**) = 50 μm, (**c**–**e**) = 5 μm.

***Strigula pycnoradians*** S.H. Jiang, J.C. Wei & Lücking, **sp. nov.** (Figure 13).

MycoBank MB 838169.

*Etymology*: Epithet of the new species “pycnoradians” is a Latin word, and conveys the more striking radiating pycnidia.

*Typus*: THAILAND, Nakorn Nayok, Wang Takrai Park, N 14°13′15″, E 101°23′34″, alt. 120 m, on living leaves, 22 August 2017, W.C. Wang WWC342_1 (HMAS–L0139611, holotype; ITS Genbank No. MW344257). 

*Diagnosis*: *Strigula pycnoradians* is closely related and morphologically similar to *S. acuticonidiarum*, but can be distinguished by the shorter asci and smaller ascospores. 

*Description*: Thallus subcuticular, dispersed into rounded to partly confluent patches, 1–7 mm across and 15–65 μm thick, with more or less an entire margin, bright to dark green. Photobiont a species of *Cephaleuros*, cells angular-rounded, 5–15 × 4–10 μm. Perithecia immersed-erumpent, hemispherical, 0.2–0.35 mm diameter and 85–115 μm high, dark green due to a thallus cover but uppermost part often exposed and black. Excipulum prosoplectenchymatous, 7.5–12.5 μm thick, colourless to brown. Involucrellum carbonaceous, 12.5–37.5 μm thick, black. Paraphyses unbranched, c. 1–2 μm thick. Asci obclavate, 32–38 × 5–10 μm. Ascospores 8 per ascus, biseriate, fusiform, 1-septate but often appearing three-septate due to oil droplets when fresh, with slight constriction at the septum, 10–12.5 × 2.5–3.5 μm, 3–4 times as long as broad. Pycnidia producing macroconidia numerous, black, wart-shaped, clustered or in radiating rows, 0.1–0.15 mm in diameter. Macroconidia one-septate, 15–20 × 2.5–4 μm, with large mucilaginous cap at the distal end. Microconidia not seen. 

*Chemistry*: No substances detected by TLC. 

*Habitat and distribution*: The new species was found in humid, semi-exposed tropical forest in Thailand. 

*Additional specimens examined*: THAILAND, Nakorn Nayok, Wang Takrai Park, N 14°13′15″, E 101°23′34″, alt. 120 m, 22 August 2017, W.C. Wang WWC353_1 (HMAS–L0139615), WWC360_1 (HMAS–L0141583). 

*Notes*: The new species is mostly similar to the closely related *Strigula acuticonidiarum* S.H. Jiang, X.L. Wei & J.C. Wei [49]. Both share the general morphology of somewhat grouped or confluent pycnidia also seen in the neotropical *S. antillarum*, which is, however, phylogenetically separate (Figure 1). *Strigula pycnoradians* differs from *S. acuticonidiarum* in the often radiating rows of pycnidia and the larger mucilaginous cap of the macroconidia. In addition, the ascospores are much smaller (12.5–20 × 3.7–5 µm in *S. acuticonidiarum*), leading also to much shorter asci (50–65 × 8–12 µm in *S. acuticonidiarum* [49]). The radiating pycnidia are reminiscent of those of *S. novae-zelandiae* (Nag Raj) Sérus. and *S. oleistrata* M. Ford, D.J. Blanchon & de Lange, both known from New Zealand [73]. However, in these two species, the macroconidia are shorter (7–15 μm) and the thalli are typically much larger.

**Figure 14:**
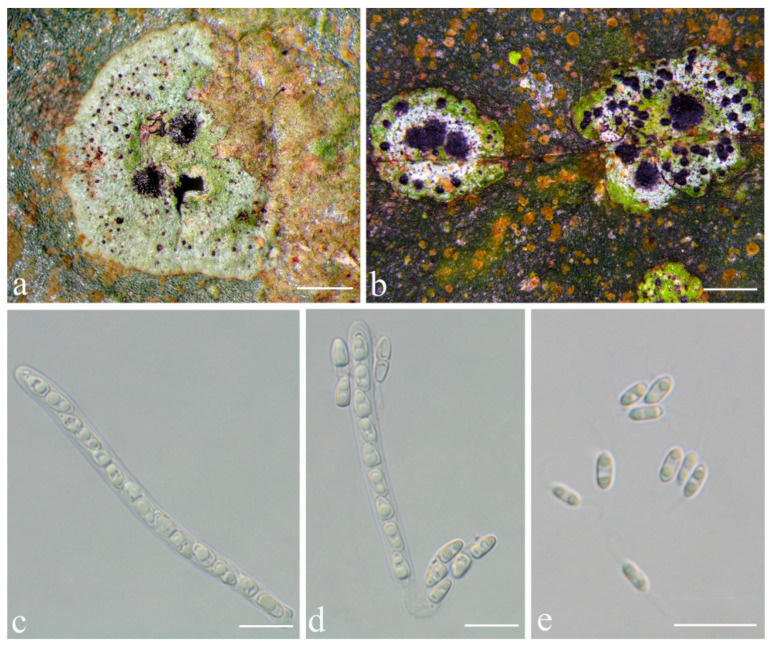
*Strigula sinoconcreta* sp. nov. (holotype, HMAS–L0139630). (**a**,**b**) Thallus; (**c**,**d**) asci and ascospores; (**e**) macroconidia. Scale bars: (**a**) = 300 μm, (**b**) = 500 μm, (**c**–**e**) = 10 μm.

***Strigula sinoconcreta*** S.H. Jiang, J.C. Wei & Lücking, **sp. nov.** (Figure 14).

MycoBank MB 838170.

*Etymology*: The epithet “sinoconcreta” refers to the region of the type material and the similarity with *Strigula concreta*. 

*Typus*: CHINA, Hainan, Changjiang county, Bawangling National Nature Reserve, N 19°04′57″, E 109°07′29″, alt. 550 m, on living leaves, 5 September 2017, S.H. Jiang HN20171454 (HMAS–L0139630, holotype; ITS Genbank No. MW344261). 

*Diagnosis*: The new species differs from *Strigula concreta* in the bright green thallus and the larger, less prominent, basally spreading perithecia. 

*Description*: Thallus subcuticular, continuous or dispersed into rounded to irregular, partly confluent patches, typically with crenulate or shortly lobulate margins, pale greyish green to green, 1.5–5 mm across and 10–15 μm thick, sometimes occurring along the nerves. Photobiont a species of *Cephaleuros*, cells 8–14 × 4–6 μm. Perithecia wart-shaped to hemispherical, basally spreading, 0.4–0.7 mm diameter and 100–180 μm high, black. Excipulum prosoplectenchymatous, 7.5–12.5 μm thick, colourless to brown. Involucrellum carbonaceous, 50–75 μm thick, black. Paraphyses unbranched, thin. Asci cylindrical, 52.5–62.5 × 5–7.5 μm. Ascospores eight per ascus, uniseriate, ellipsoid, one-septate, with distinct constriction at septum and often broken into halves outside asci, 9–12.5 × 2–3 μm, 3–4 times as long as broad. Pycnidia wart-shaped, those producing macroconidia 0.1–0.15 mm, those producing microconidia 0.05–0.1 mm diameter, black. Macroconidia bacillar, non-septate, 4–5 × 1.5–2.5 μm, with appendage at one end or both ends c. 5–10 μm long. Microconidia fusiform, aseptate, 4–5 × 1.5–2 μm. 

*Chemistry*: No substances detected by TLC. 

*Habitat and distribution*: The new species was discovered in humid forest in southern China. Individuals mostly grew on living leaves with a rigid texture. 

*Additional specimens examined*: CHINA, Hainan, Changjiang county, Bawangling National Nature Reserve, N 19°04′57″, E 109°07′29″, alt. 550 m, on living leaves, 5 September 2017, S.H. Jiang HN20171477 (HMAS–L0139631). 

*Notes*: *Strigula sinoconcreta* agrees with *S. concreta* in general habit and in the uniseriate, short ascospores partly breaking into halves, but can be recognized by the bright green thallus (grey–green in *S. concreta* [45]) and the larger, flatter, basally somewhat spreading perithecia. The new species was phylogenetically distinct from *S. intermedia* (Figure 1 and Figure 2) and differs from the latter in the thicker thallus not bordered by black lines.

**Figure 15:**
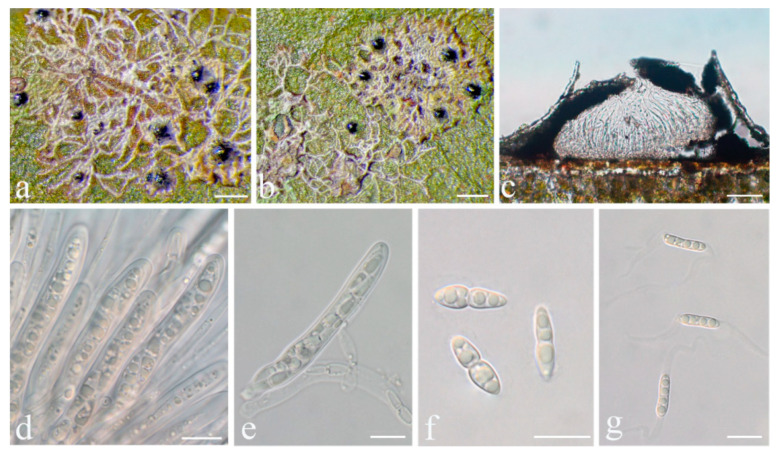
*Strigula stenoloba* sp. nov. (holotype, HMAS–L0139622). (**a**,**b**) Thallus; (**c**) perithecia; (**d**,**e**) asci; (**f**) ascospores; (**g**) macroconidia. Scale bars: (**a**) = 600 μm, (**b**) = 500 μm, (**c**) = 50 μm, (**d**–**g**) = 10 μm.

***Strigula stenoloba*** S.H. Jiang, J.C. Wei & Lücking, **sp. nov.** (Figure 15).

MycoBank MB 838171.

*Etymology*: The epithet “stenoloba” refers to the thin, meandering lobes. 

*Typus*: CHINA, Hunan, Chenzhou city, Mangshan National Nature Reserve, N 24°56′59″, E 112°56′02″, alt. 1300 m, on living leaves, 13 September 2017, S.H. Jiang HN20170463 (HMAS–L0139622, holotype). 

*Diagnosis*: *Strigula stenoloba* differs from other species in the genus by its very narrow, irregular lobes. 

*Description*: Thallus subcuticular, continuous, occurring or not along the nerves, margins, and scars of leaves, formed of dichotomously branched, narrow, convex lobes that can fuse laterally and form a reticulate pattern, margins crenulate, 3–7 mm across and 10–15 μm thick. Photobiont a species of *Cephaleuros*, cells angular-rounded, 8–13 × 4–7 μm. Perithecia covered by thin, thallus layer up to ostiole, wart-shaped, 0.4–0.6 mm diameter and 150–230 μm high, greyish to greenish black. Excipulum prosoplectenchymatous, 5–7.5 μm thick, colourless to brown. Involucrellum carbonaceous, 35–50 μm thick, black. Paraphyses unbranched, thin. Asci obclavate, 50–75 × 7.5–10 μm. Ascospores eight per ascus, biseriate, fusiform-ellipsoid to fusiform, with acute or rather rounded ends, one-septate, with constriction at septum, 12.5–17.5 × 3–5 μm. Pycnidia exposed, wart-shaped, black and shiny, those producing macroconidia 0.1–0.2 mm, those producing microconidia 0.07–0.15 mm diameter. Macroconidia bacillar, one-septate, 12.5–15 × 2.5–3.7 μm, with 17–35 μm long appendage at both ends that may be terminated by a hook. Pycnidia producing microconidia not seen. 

*Chemistry*: No substances detected by TLC. 

*Habitat and distribution*: The new species was found on living leaves in humid, semi-exposed forest habitats in southern China. 

*Additional specimens examined*: CHINA, Hunan, Chenzhou city, Mangshan National Nature Reserve, N 24°56′59″, E 112°56′02″, alt. 1300 m, on living leaves, 13 September 2017, S.H. Jiang HN20170451 (HMAS–L0139621). 

*Notes*: Unfortunately, we were unable to obtain molecular data for this material. Yet, the morphological features are so unique that we decided to describe it formally as a new species. At first glance, one could consider this a barely lichenised form or an early ontogenetic stage, but the morphology was consistent across all individuals seen in the type collection and different from anything we have observed in other species. The formation of fully mature perithecia also contradicts the interpretation of this material as an aberrant form of a known species. The new species is characterised by its very thin, widely separated, partly anastomosing lobes. It should not be confused with *S. delicata* Sérus., a species described from New Zealand [74]. The latter has a much better developed thallus with narrow but regular lobes, although it agrees in ascospore and macroconidial size. Species of *Racoplaca* also differ in the regular thallus lobes, which are flatter and typically olive-green to brownish [45]. Somewhat similar is *R. tremens* (Müll. Arg.) S.H. Jiang, Lücking & J.C. Wei, known from Brazil, which agrees in the somewhat irregular, narrow, more or less free laciniae, but the laciniae are completely flat and bordered by a thin, black line, the ascospores are longer (17–23 × 3–5 μm), and the involucrellum is thinner (15–25 μm thick); macroconidia are unfortunately not known [45].

**Figure 16:**
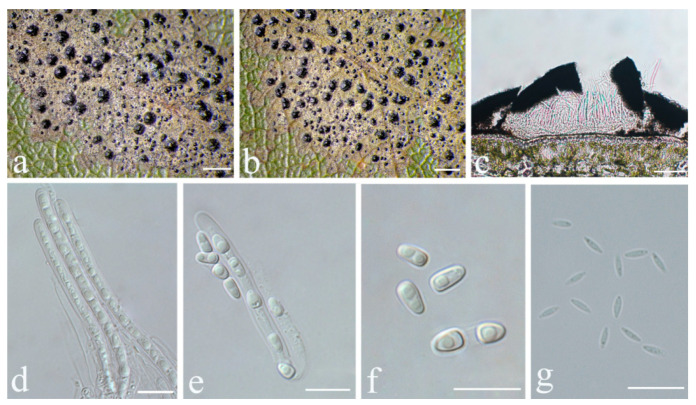
*Strigula subtilissimoides* sp. nov. (**a**,**b**) Thallus (holotype, HMAS–L0139253); (**c**) perithecia (HMAS–L0139255); (**d**) asci (holotype, HMAS–L0139253); (**e**,**f**) ascospores (holotype, HMAS–L0139253); (**g**) microconidia (HMAS–L0139255). Scale bars: (**a**–**c**) = 500 μm, (**d**–**g**) = 10 μm.

***Strigula subtilissimoides*** S.H. Jiang, J.C. Wei & Lücking, **sp. nov.** (Figure 16).

MycoBank MB 838172.

*Etymology*: The thallus feature of this species is its similarity to *Racoplaca subtilissima*, although not related to the latter, so we decided to name it *Strigula subtilissimoides*.

*Typus*: CHINA, Guangxi, Shangsi County, Shiwan Mountain Forest Park, N 21°54′13″, E 107°54′13″, alt. 264 m, on living leaves, 5 December 2015, S.H. Jiang GX201511373 (HMAS–L0139253, holotype; ITS Genbank No. MK206352).

*Diagnosis*: *Strigula subtilissimoides* is intermediate between *S. maculata* and *Racoplaca subtilissima* in thallus, but the ascospores belong to the *S. nitidula* type. Phylogenetically different from the other *S. nitidula* clade. 

*Description*: Thallus subcuticular, 5–25 mm across and 12.5–27.5 μm thick, with entire to crenulate margins to irregularly laciniate, pale to dark olive to brownish, sometimes with tiny verrucae on the surface, nitidous. Photobiont a species of *Cephaleuros*, cells angular-rounded, 8–12 × 4–6 μm. Perithecia exposed, conical to wart-shaped, 0.4–0.6 mm diameter and 120–200 μm high, black. Excipulum prosoplectenchymatous, 12.5–22.5 μm thick, brown. Involucrellum 22–60 μm thick, carbonaceous, black. Paraphyses unbranched and thin. Asci cylindrical or thread like, numerous and dense, 40–90 × 3–5 μm. Ascospores eight per ascus, uniseriate, ellipsoid, one-septate, sometimes with 1–2 oil droplets per cell when fresh, with distinct constriction at the septum and sometimes broken into halves outside the asci, 8.5–12.5 × 1.5–2.5 μm. Pycnidia producing macroconidia not seen. Pycnidia producing microconidia wart-shaped, 0.05–0.1 mm diameter, black. Microconidia fusiform, aseptate, 4–5 × 1.5–2 μm.

*Chemistry*: No substances detected by TLC. 

*Habitat and distribution*: The new species was found on living leaves in humid, semi-exposed forest in southern China. 

*Additional specimens examined*: CHINA, Guangxi, Shangsi County, Shiwan Mountain Forest Park, N 21°54′13″, E 107°54′13″, alt. 264 m, on living leaves, 5 December 2015, S.H. Jiang GX201511376 (HMAS–L0139293), GX201511384 (HMAS–L0139254), GX201511392 (HMAS–L0139256), GX201511393 (HMAS–L0139255); N 21°54′03″, E 107°54′26″, alt. 342 m, on living leaves, 25 May 2015, X.L. Wei & J.H. Wang GX20150270 (HMAS–L0139277); Hainan, Ledong County, Jianfengling, Mingfenggu, N 18°44′5″, E 108°52′8″, alt. 920 m, on living leaves, 11 December 2014, J.H. Wang & R.D. Liu HN2014149 (HMAS–L0130556), HN2014149_2 (HMAS–L0141629), J.H. Wang & R.D. Liu HN2014160_3 (HMAS–L0141599), J.H. Wang & R.D. Liu HN2014240_2 (HMAS–L0141389); 27 July 2009, J.C. Wei WJC034f11 (HMAS–L0139204). 

*Notes*: *Strigula subtilissimoides* is another new species besides *S. pseudosubtilissima* (see above) that is morphologically similar to *Racoplaca subtilissima*. In this case, the resemblance is even more striking, and superficial examination may place this material under the latter species. However, the more or less exposed, black perithecia, and particularly the uniseriate, cylindrical asci with short ascospores breaking into halves reveal a close relationship with *S. nitidula*, which is supported by the molecular data, which also show that *S. pseudosubtilissima* and *S. subtilissimoides* are phylogenetically distinct (Figure 1 and Figure 2). Morphologically, both differ chiefly in thallus colour. *Strigula subtilissimoides* is sister to *S. sinoaustralis* S.H. Jiang, X.L. Wei & J.C. Wei, another taxon with *S. nitidula* morphology but which features numerous small, white papillae on the thallus surface [48].

## 4. Discussion

At first glance, the discovery of so many new species in the foliicolous genera *Strigula* and *Racoplaca* may seem surprising. However, based on Santesson’s (1952) monograph, the species concept in foliicolous lichens has been comparatively broad, with many species presumably widespread, pantropical, or even subcosmopolitan [75]. This concept has only recently been replaced with more fine-scaled species delimitation in many foliicolous lineages [45]. However, the subcuticularly growing species of *Strigula* have so far been considered morphologically variable, under the assumption that leaf characteristics affect thallus shape [45]. Indeed, molecular data support this view [50]. At the same time, phylogenetic studies, including the present one, have revealed a great deal of cryptic speciation in these lichen-forming fungi [48,49,50,73]. Given that these studies are partly based on multiple markers (ITS, nuSSU, nuLSU, TEF1-α, RPB2), such revised, much finer species concepts reflect the reality much better than previous, morphology-based concepts, as well as because the newly recognized cryptic lineages often emerge on long-stem branches and are not even directly related. Our data also show that ITS alone provides high resolution in *Strigulaceae* at the species level and the corresponding topology is highly congruent with that of other markers, making the ITS a promising single marker for a broad molecular screening in this group.

The notion that the currently available molecular data for this enigmatic group of lichen-forming fungi are almost exclusively from continental Southeast Asia, at the northern border of the tropical belt, and very few or no such data exist for the Neotropics, the African Paleotropics, the Indian subcontinent, the Indopacific, and Australasia, suggests that species richness in foliicolous *Strigulaceae*, in particular *Strigula* s.str., is grossly underestimated. Taking into account the presently available data, with 19 new species recognised based on molecular phylogenies from China, South Korea, Thailand, and New Zealand, and several other species reinstated from previous synonymy [48,49,50,73,76], we anticipate that additional cryptic species will be discovered in these widespread collective taxa in other regions, particularly in *Phylloporis obducta*, *P. phyllogena*, *Puiggariella nemathora*, *Racoplaca maculata*, *R. subtillissima*, *Strigula antillarum*, *S. concreta*, *S. nitidula*, *S. smaragdula*, and *S. subelegans*. In the hitherto studied species, based on the aforementioned and the present study, the factor of hidden diversity in presumably known species ranges from twofold (e.g., *Racoplaca maculata*, *R. melanobapha*; *Strigula microspora*, *S. nitidula*) to threefold (e.g., *Puiggariella nemathora*, *R. subtilissima*, *Strigula concreta*) to up to fivefold (*S. antillarum*, *S. smaragdula*), for a weighted mean of a threefold increase. These findings align well with Lücking et al. [36], who also showed multiplication factors between two and five in studied species complexes. Given that the data on *Strigulaceae* are mostly from Southeast Asia, even higher multiplication factors (a weighted mean of up to five) could be assumed when expanding such studies to tropical America and Africa. If we consider the currently known 67 species in this clade of six genera, assume that about one third of these represent species complexes with hidden diversity, and apply the factor five to them, the total species richness in this group can be extrapolated at close to 150 species. This estimate may still be conservative, given that the high levels of cryptic or near-cryptic speciation thus far revealed are concentrated within a narrow region in Southeast Asia. Our current findings, thus, appear to be just the proverbial tip of the iceberg, the actual size of which is difficult to assess.

Our results also put into perspective the recently proposed split of *Strigula* s.lat. into seven genera, namely *Phyllocharis*, *Phylloporis*, *Puiggariella*, *Raciborskiella*, *Racoplaca*, *Serusiauxiella*, and *Strigula* [41,47]. One may consider this an attempt at oversplitting. However, as explained by Jiang et al. [41,47], the newly recognised or reinstated genera not only emerge mostly on long-stem branches, indicating a longer time of separate evolution, but also correlate well with various phenotype characteristics. With an estimated total of at least 150 species in these genera overall, the species:genus ratio would result in a little over 20:1, which corresponds well to the overall ratio in lichenised fungi, and fungi and plants in general [46,77], while genera in animals are, on average, less than half this size [77].

## Data Availability

Publicly available datasets were analysed in this study. All resulting alignments were deposited in TreeBASE (http://www.treebase.org (accessed on 12 October 2021); accession number S27476). All newly generated sequences were deposited in GenBank (https://www.ncbi.nlm.nih.gov/genbank/ (accessed on 12 October 2021); Table S1). All new taxa were deposited in MycoBank (https://www.mycobank.org/ (accessed on 12 October 2021)).

## Supplementary Materials

The following are available online at https://www.mdpi.com/article/10.3390/jof8010002/s1, Table S1: Specimens and sequences for phylogenetic analysis; Table S2: Comparison between *Racoplaca macrospora* and *Racoplaca maculatoides*; Figure S1: Phylogenetic tree constructed through ML analyses based on the ITS for *Racoplaca*.
