# Supplementary material for: Twelve New Species Reveal Cryptic Diversification in Foliicolous Lichens of Strigula s.lat. (Strigulales, Ascomycota)
Source: J Fungi (Basel). 2021 Dec 21;8(1):2. doi: 10.3390/jof8010002 (PMC8781847; doi:10.3390/jof8010002)
Supplement: Supplementary file 1 [file jof-08-00002-s001.zip › Table S1.pdf]

Table S1 Specimens and sequences for phylogenetic analysis

**Species**	**Specimen No.**	**Country**	**Collector**	**ITS**	**nuLSU**	**nuSSU**	***TEF1-α***	***RPB2***
Flavobathelium epiphyllum	MPN67				GU327717	JN887382	JN887423	—
Phyllobathelium anomalum	MPN242				GU327722	JN887386	JN887430	—
Phylloporis cf. *obducta*	**HMAS-L0139218**	China	S.H. Jiang	**MW344124**	MN720034	MN727002	MN738504	MN738529
P. cf. *obducta*	**HMAS-L0139222**	China	S.H. Jiang	**MW344125**				
P. cf. *obducta*	**HMAS-L0139237**	China	S.H. Jiang	**MW344126**				
P. cf. *obducta*	**HMAS-L0139246**	China	S.H. Jiang	**MW344127**				
P. cf. *obducta*	**HMAS-L0139247**	China	S.H. Jiang	**MW344128**	MN720035	MN727003	MN738505	MN738530
P. cf. *obducta*	**HMAS-L0139251**	China	S.H. Jiang	**MW344129**				
P. cf. *obducta*	**HMAS-L0139282**	China	S.H. Jiang	**MW344130**				
P. cf. *obducta*	**HMAS-L0139284**	China	S.H. Jiang	**MW344131**				
P. cf. *obducta*	**HMAS-L0139286**	China	S.H. Jiang	**MW344132**	MN720036	MN727004	MN738506	MN738531
P. cf. *obducta*	**HMAS-L0139324**	China	S.H. Jiang	**MW344133**				
P. cf. *obducta*	**HMAS-L0139339**	China	X.L. Wei & S.H. Jiang	**MW344134**	MN720037	MN727005	MN738507	MN738532
P. cf. *obducta*	**HMAS-L0139635**	China	S.H. Jiang	**MW344135**	MN720038	MN727006	MN738508	MN738533
P. palmae	**ISE-33374**	Brazil	R. Lücking et al.	**MW344136**				
P. palmae	**ISE-33667**	Brazil	R. Lücking et al.	**MW344137**				
P. palmae	**ISE-33670**	Brazil	R. Lücking et al.	**MW344138**				
P. phyllogena	**ISE-33944**	Brazil	R. Lücking et al.	**MW344139**	MN720039	MN727007	MN738509	MN738534
Puiggariella confluens	**B600205040**	Guatemala	R. Lücking et al.	**MW344140**				
Pui. confluens	**B600205073**	Costa Rica	R. Lücking et al.	**MW344141**				
Pui. confluens	**B600205074**	Costa Rica	R. Lücking et al.	**MW344142**				
Pui. nemathora	**ISE-32586**	Brazil	R. Lücking et al.	**MW344143**	—	MN727009	MN738510	—
Pui. nemathora	**ISE-32794**	Brazil	R. Lücking et al.	**MW344144**	—	MN727008	—	MN738535
Pui. nigrocincta	**HMAS-L0139188**	China	X.Y. Liu	**MW344145**				
Pui. nigrocincta	**HMAS-L0139194**	China	X.Y. Liu	**MW344146**				
Pui. nigrocincta	**HMAS-L0139287**	China	S.H. Jiang	**MW344147**				
Pui. nigrocincta	**HMAS-L0139335**	China	X.L. Wei & S.H. Jiang	**MW344148**	MN720040	MN727010	MN738511	MN738536
Pui. nigrocincta	**HMAS-L0139350**	China	X.L. Wei & S.H. Jiang	**MW344149**	MN720041	MN727011	MN738512	MN738537
Pui. nigrocincta	**HMAS-L0139360**	China	X.L. Wei & S.H. Jiang	**MW344150**				
Pui. nigrocincta	**HMAS-L0139366**	China	X.L. Wei & S.H. Jiang	**MW344151**				
Pui. nigrocincta	**HMAS-L0139368**	China	X.L. Wei & S.H. Jiang	**MW344152**				
Pui. nigrocincta	**HMAS-L0139370**	China	X.L. Wei & S.H. Jiang	**MW344153**				

Pui. nigrocincta	**HMAS-L0141541**	China	X.L. Wei & S.H. Jiang	**MW344154**	MN720042	MN727012	MN738513	MN738538
Pui. nigrocincta	**HMAS-L0141544**	China	X.L. Wei & S.H. Jiang	**MW344155**				
Pui. nigrocincta	**HMAS-L0141549**	China	X.L. Wei & S.H. Jiang	**MW344156**				
Raciborskiella janeirensis	**HMAS-L0130589**	China	J.H. Wang & R.D. Liu	**MW344157**	MN720043	MN727013	MN738514	—
Raci. janeirensis	**HMAS-L0137672**	China	J.C. Wei	**MW344158**				
Raci. talaumae	**HMAS-L0139624**	China	S.H. Jiang	**MW344159**	MN720044	MN727014	MN738515	—
Raci. talaumae	**HMAS-L0139625**	China	S.H. Jiang	**MW344160**				
Raci. talaumae	**HMAS-L0139626**	China	S.H. Jiang	**MW344161**				
Raci. talaumae	**HMAS-L0139627**	China	S.H. Jiang	**MW344162**	MN720045	MN727015	MN738516	—
Raci. talaumae	**HMAS-L0139628**	China	S.H. Jiang	**MW344163**				
Raci. talaumae	**HMAS-L0139629**	China	S.H. Jiang	**MW344164**				
Racoplaca macrospora sp. nov.	**HMAS-L0139207**	China	X.L. Wei & J.H. Wang	**MW344165**				
R. macrospora sp. nov.	**HMAS-L0139274**	China	X.L. Wei & J.H. Wang	**MW344166**				
R. macrospora sp. nov.	**HMAS-L0139327**	China	X.L. Wei & J.H. Wang	**MW344167**				
R. macrospora sp. nov.	**HMAS-L0141626**	China	J.H. Wang & R.D. Liu	**MW344168**				
R. maculatoides sp. nov.	**HMAS-L0130558**	China	J.H. Wang & R.D. Liu	**MW344169**				
R. maculatoides sp. nov.	**HMAS-L0130601**	China	J.H. Wang & R.D. Liu	**MW344170**				
R. maculatoides sp. nov.	**HMAS-L0139159**	China	X.L. Wei & J.H. Wang	**MW344171**				
R. maculatoides sp. nov.	**HMAS-L0139162**	China	X.L. Wei & J.H. Wang	**MW344172**	**MW357058**	**MW375713**	**MW346600**	**MW346612**
R. maculatoides sp. nov.	**HMAS-L0139164**	China	X.L. Wei & J.H. Wang	**MW344173**	**MW357059**	**MW375714**	**MW346601**	**MW346613**
R. maculatoides sp. nov.	**HMAS-L0139170**	China	X.L. Wei & J.H. Wang	**MW344174**				
R. maculatoides sp. nov.	**HMAS-L0141589**	China	X.L. Wei & J.H. Wang	**MW344175**				
R. maculatoides sp. nov.	**HMAS-L0141590**	China	X.L. Wei & J.H. Wang	**MW344176**				
R. maculatoides sp. nov.	**HMAS-L0141595**	China	J.H. Wang & R.D. Liu	**MW344177**				
R. maculatoides sp. nov.	**HMAS-L0141602**	China	J.H. Wang & R.D. Liu	**MW344178**				
R. maculatoides sp. nov.	**HMAS-L0141613**	China	J.H. Wang & R.D. Liu	**MW344179**				
R. maculatoides sp. nov.	**HMAS-L0141616**	China	J.H. Wang & R.D. Liu	**MW344180**				
R. melanobapha	**HMAS-L0139173**	China	X.L. Wei & J.H. Wang	**MW344181**				
R. melanobapha	**HMAS-L0139275**	China	X.L. Wei & J.H. Wang	**MW344182**				
R. melanobapha	**HMAS-L0139328**	China	X.L. Wei & J.H. Wang	**MW344183**				
R. melanobapha	**HMAS-L0139331**	China	X.L. Wei & J.H. Wang	**MW344184**	MN720046	MN727016	MN738517	MN738539
R. melanobapha	**HMAS-L0139623**	China	S.H. Jiang	**MW344185**	MN720047	MN727017	MN738518	MN738540
R. melanobapha	**HMAS-L0141593**	China	X.L. Wei & J.H. Wang	**MW344186**				
R. subtilissima	**ISE-32602**	Brazil	R. Lücking et al.	**MW344187**	—	MN727018	MN738519	—
Serusiauxiella filifera	HMAS-L0130625			MN720009				
Ser. filifera	HMAS-L0130627			MN720010				

Ser. filifera	HMAS-L0130629			MN720011	MN720048	MN727019	MN738520	MN738541
Ser. filifera	HMAS-L0139219			MN720019				
Ser. filifera	HMAS-L0139223			MN720020				
Ser. filifera	HMAS-L0139234			MN720022				
Ser. filifera	HMAS-L0139270			MN720012				
Ser. filifera	HMAS-L0139272			MN720013				
Ser. filifera	HMAS-L0139288			MN720021				
Ser. filifera	HMAS-L0139298			MN720015				
Ser. filifera	HMAS-L0141639			MN720014				
Ser. filifera	HMAS-L0141641			MN720016				
Ser. filifera	HMAS-L0141644			MN720017				
Ser. filifera	HMAS-L0141648			MN720018	MN720049	MN727020	MN738521	MN738542
Ser. filifera	HMAS-L0141656			MN720023	MN720050	MN727021	MN738522	MN738543
Ser. filifera	HMAS-L0141660			MN720024				
Ser. flagellata	HMAS-L0130554			MN720025				
Ser. flagellata	HMAS-L0139216			MN720027				
Ser. flagellata	HMAS-L0141609			MN720026				
Ser. sinensis	HMAS-L0141605			MN720033				
Ser. sinensis	HMAS-L0141611			MN720031	MN720053	MN727022	MN738525	MN738546
Ser. sinensis	HMAS-L0141612			MN720032				
Ser. sinensis	HMAS-L0141614			MN720030				
Strigula acuticonidiarum	HMAS-L0138045			KY100290	MK206236	MK206217	MK273083	MK273111
S. acuticonidiarum	HMAS-L0138046			KY100295				
S. acuticonidiarum	HMAS-L0138047			KY100296				
S. acuticonidiarum	HMAS-L0138048			KY100291				
S. acuticonidiarum	HMAS-L0138049			KY100292				
S. acuticonidiarum	HMAS-L0138050			KY100293				
S. acuticonidiarum	HMAS-L0138051			KY100294				
S. albomaculata	**HMAS-L0139202**	Cambodia	X.L. Wei	**MW344188**				
S. antillarum	**B600205079**	Cuba	R. Lücking et al.	**MW344189**				
S. antillarum	**B600205080**	Cuba	R. Lücking et al.	**MW344190**				
S. antillarum	**B600205082**	Cuba	R. Lücking et al.	**MW344191**				
S. antillarum	**B600205084**	Cuba	R. Lücking et al.	**MW344192**	MN720054	—	MN738526	MN738547
S. cf. *atrocarpoides*	**HMAS-L0139342**	China	X.L. Wei & S.H. Jiang	**MW344194**	MN720055	MN727023	MN738527	MN738548
S. cf. *atrocarpoides*	**HMAS-L0139346**	China	X.L. Wei & S.H. Jiang	**MW344195**				
S. cf. *atrocarpoides*	**HMAS-L0139180**	China	X.Y. Liu	**MW344193**				

S. cf. *atrocarpoides*	**HMAS-L0141560**	China	X.L. Wei & S.H. Jiang	**MW344196**				
S. cf. *macaronesica*	**HMAS-L0130609**	China	J.H. Wang & R.D. Liu	**MK206313**				
S. cf. *macaronesica*	**HMAS-L0130611**	China	J.H. Wang & R.D. Liu	**MK206314**				
S. cf. *macaronesica*	**HMAS-L0130615**	China	J.H. Wang & R.D. Liu	**MK206315**	MK206251	MK206230	MK273098	MK273126
S. cf. *macaronesica*	**HMAS-L0139168**	China	X.L. Wei & J.H. Wang	**MK206317**				
S. cf. *macaronesica*	**HMAS-L0139206**	China	J.C. Wei	**MK206318**				
S. cf. *macaronesica*	**HMAS-L0139208**	China	J.C. Wei	**MK206319**				
S. cf. *macaronesica*	**HMAS-L0139258**	China	S.H. Jiang	**MK206321**				
S. cf. *macaronesica*	**HMAS-L0139260**	China	S.H. Jiang	**MK206322**	MK206252	MK206231	MK273099	MK273127
S. cf. *macaronesica*	**HMAS-L0139261**	China	S.H. Jiang	**MK206323**				
S. cf. *macaronesica*	**HMAS-L0139262**	China	S.H. Jiang	**MK206324**				
S. cf. *macaronesica*	**HMAS-L0139330**	China	X.L. Wei & J.H. Wang	**MW344197**				
S. cf. *macaronesica*	**HMAS-L0141580**	China	X.L. Wei & J.H. Wang	**MW344198**				
S. cf. *macaronesica*	**HMAS-L0141581**	China	X.L. Wei & J.H. Wang	**MW344199**				
S. cf. *macaronesica*	**HMAS-L118983**	China	J.C. Wei & W. Guo	**MK206308**				
S. cf. *smaragdula*	HMAS-L0130621			KY100298				
S. cf. *smaragdula*	HMAS-L0138067			KY100300				
S. cf. *smaragdula*	HMAS-L0138068			KY100299				
S. cf. *smaragdula*	HMAS-L0139166			KY100296	MK206235	MK206216	MK273082	MK273110
S. cf. *smaragdula*	**HMAS-L0141395**	China	X.L. Wei & S.H. Jiang	**MK206357**	MK206234	MK206215	MK273081	MK273109
S. cf. *smaragdula*	**HMAS-L0141396**	China	X.L. Wei & J.H. Wang	**MK206359**	MK206233	MK206214	MK273080	MK273108
S. dispersa	HMAS-L0137212			KX216701	MK206250	—	MK273097	MK273125
S. dispersa	HMAS-L0137213			KX216700				
S. guangdongensis sp. nov.	**HMAS-L0139158**	China	X.L. Wei & J.H. Wang	**MW344200**				
S. guangdongensis sp. nov.	**HMAS-L0139161**	China	X.L. Wei & J.H. Wang	**MW344201**				
S. guangdongensis sp. nov.	**HMAS-L0139169**	China	X.L. Wei & J.H. Wang	**MW344202**	**MW357060**	**MW375715**	**MW346602**	—
S. guangxiensis	HMAS-L0138040			KY100301	MK206256	—	MK273103	MK273131
S. guangxiensis	HMAS-L0138041			KY100304	MK206257	—	MK273104	MK273132
S. guangxiensis	HMAS-L0138042			KY100305				
S. guangxiensis	HMAS-L0138044			KY100302				
S. intermedia sp. nov.	**HMAS-L0139176**	China	X.Y. Liu	**MK206304**	**MK206254**	—	**MK273101**	**MK273129**
S. intermedia sp. nov.	**HMAS-L0139177**	China	X.Y. Liu	**MK206305**				
S. intermedia sp. nov.	**HMAS-L0139178**	China	X.Y. Liu	**MK206306**	**MK206255**	—	**MK273102**	**MK273130**
S. laevis sp. nov.	**HMAS-L0139172**	China	X.L. Wei & J.H. Wang	**MK206348**				
S. laevis sp. nov.	**HMAS-L0139174**	China	X.L. Wei & J.H. Wang	**MK206349**				
S. laevis sp. nov.	**HMAS-L0141592**	China	X.L. Wei & J.H. Wang	**MW344203**	**MK206253**	**MK206232**	**MK273100**	**MK273128**

S. laevis sp. nov.	**HMAS-L0141594**	China	X.L. Wei & J.H. Wang	**MW344204**				
S. macrocarpa	HMAS-L0137662			KY083728				
S. macrocarpa	HMAS-L0137663			KY083729				
S. macrocarpa	HMAS-L0137664			KY083731				
S. macrocarpa	HMAS-L0137666			KY083730				
S. macrocarpa	**HMAS-L0139289**	China	S.H. Jiang	**MK206341**	MK206241	MK206222	MK273088	MK273116
S. macrocarpa	**HMAS-L0141394**	China	S.H. Jiang	**MK206326**	MK206240	MK206221	MK273087	MK273115
S. microcarpa sp. nov.	**HMAS-L0139191**	China	X.Y. Liu	**MW344205**	**MW357061**	**MW375716**	**MW346603**	**MW346614**
S. microcarpa sp. nov.	**HMAS-L0139192**	China	X.Y. Liu	**MW344206**				
S. microcarpa sp. nov.	**HMAS-L0139193**	China	X.Y. Liu	**MW344207**				
S. microcarpa sp. nov.	**HMAS-L0139196**	China	X.Y. Liu	**MW344208**	**MW357062**	**MW375717**	**MW346604**	**MW346615**
S. microcarpa sp. nov.	**HMAS-L0139199**	China	X.Y. Liu	**MW344209**				
S. microcarpa sp. nov.	**HMAS-L0139200**	China	X.Y. Liu	**MW344210**				
S. microcarpa sp. nov.	**HMAS-L0139201**	China	X.Y. Liu	**MW344211**				
S. microcarpa sp. nov.	**HMAS-L0141585**	China	X.Y. Liu	**MW344212**				
S. microcarpa sp. nov.	**HMAS-L0141588**	China	X.Y. Liu	**MW344213**				
S. nitidula	**B600205019**	Guatemala	R. Lücking et al.	**MW344214**				
S. nitidula	**B600205029**	Guatemala	R. Lücking et al.	**MW344215**				
S. nitidula	**B600205077**	Cuba	R. Lücking et al.	**MW344216**				
S. nitidula	**HMAS-L0139343**	China	X.L. Wei & S.H. Jiang	**MW344217**				
S. nitidula	**HMAS-L0139358**	China	X.L. Wei & S.H. Jiang	**MW344218**	MN788374	MN788375	MN793983	MN793982
S. nitidula	**HMAS-L0139361**	China	X.L. Wei & S.H. Jiang	**MW344219**				
S. nitidula	**HMAS-L0139367**	China	X.L. Wei & S.H. Jiang	**MW344220**				
S. nitidula	**HMAS-L0141586**	China	X.Y. Liu	**MW344221**				
S. nitidula	**HMAS-L0141587**	China	X.Y. Liu	**MW344222**				
S. prasina	**B600205022**	Guatemala	R. Lücking et al.	**MW344223**				
S. prasina	**B600205024**	Guatemala	R. Lücking et al.	**MW344224**				
S. prasina	**B600205026**	Guatemala	R. Lücking et al.	**MW344225**	—	MN727024	MN738528	—
S. prasina	**B600205065**	Panama	R. Lücking et al.	**MW344226**				
S. pseudoantillarum sp. nov.	**HMAS-L0130571**			KY100288	**MW357063**	**MW375718**	**MW346605**	**MW346616**
S. pseudoantillarum sp. nov.	**HMAS-L0130573**	China	J.H. Wang & R.D. Liu	KY100289	**MK206237**	**MK206218**	**MK273084**	**MK273112**
S. pseudoantillarum sp. nov.	HMAS-L0137208			KX216697				
S. pseudoantillarum sp. nov.	HMAS-L0137209			KX216696				
S. pseudoantillarum sp. nov.	HMAS-L0137211			KX216702				
S. pseudoantillarum sp. nov.	**HMAS-L0139347**	China	X.L. Wei & S.H. Jiang		**MK206239**	**MK206220**	**MK273086**	**MK273114**
S. pseudoantillarum sp. nov.	**HMAS-L0139616**	Thailand	W.C. Wang		**MW357064**	**MW375719**	**MW346606**	**MW346617**

S. pseudoantillarum sp. nov.	**HMAS-L0139618**	Thailand	W.C. Wang		**MW357065**	**MW375720**	**MW346607**	**MW346618**
S. pseudosubtilissima sp. nov.	**HMAS-L0130553**	China	J.H. Wang & R.D. Liu	**MW344227**	**MW357066**	**MW375721**	**MW346608**	**MW346619**
S. pseudosubtilissima sp. nov.	**HMAS-L0130560**	China	J.H. Wang & R.D. Liu	**MW344228**				
S. pseudosubtilissima sp. nov.	**HMAS-L0130570**	China	J.H. Wang & R.D. Liu	**MW344229**				
S. pseudosubtilissima sp. nov.	**HMAS-L0130578**	China	J.H. Wang & R.D. Liu	**MW344230**				
S. pseudosubtilissima sp. nov.	**HMAS-L0130591**	China	J.H. Wang & R.D. Liu	**MW344231**				
S. pseudosubtilissima sp. nov.	**HMAS-L0130595**	China	J.H. Wang & R.D. Liu	**MW344232**				
S. pseudosubtilissima sp. nov.	**HMAS-L0130597**	China	J.H. Wang & R.D. Liu	**MW344233**	**MW357067**	**MW375722**	**MW346609**	**MW346620**
S. pseudosubtilissima sp. nov.	**HMAS-L0130599**	China	J.H. Wang & R.D. Liu	**MW344234**				
S. pseudosubtilissima sp. nov.	**HMAS-L0130602**	China	J.H. Wang & R.D. Liu	**MW344235**				
S. pseudosubtilissima sp. nov.	**HMAS-L0130603**	China	J.H. Wang & R.D. Liu	**MW344236**				
S. pseudosubtilissima sp. nov.	**HMAS-L0130605**	China	J.H. Wang & R.D. Liu	**MW344237**				
S. pseudosubtilissima sp. nov.	**HMAS-L0130607**	China	J.H. Wang & R.D. Liu	**MW344238**				
S. pseudosubtilissima sp. nov.	**HMAS-L0130612**	China	J.H. Wang & R.D. Liu	**MW344239**				
S. pseudosubtilissima sp. nov.	**HMAS-L0139217**	China	X.Y. Liu	**MW344240**				
S. pseudosubtilissima sp. nov.	**HMAS-L0139292**	China	S.H. Jiang	**MW344241**				
S. pseudosubtilissima sp. nov.	**HMAS-L0139329**	China	X.L. Wei & J.H. Wang	**MW344242**				
S. pseudosubtilissima sp. nov.	**HMAS-L0139633**	China	S.H. Jiang	**MW344243**				
S. pseudosubtilissima sp. nov.	**HMAS-L0141582**	China	X.L. Wei & J.H. Wang	**MW344244**				
S. pseudosubtilissima sp. nov.	**HMAS-L0141596**	China	J.H. Wang & R.D. Liu	**MW344245**				
S. pseudosubtilissima sp. nov.	**HMAS-L0141598**	China	J.H. Wang & R.D. Liu	**MW344246**				
S. pseudosubtilissima sp. nov.	**HMAS-L0141603**	China	J.H. Wang & R.D. Liu	**MW344247**				
S. pseudosubtilissima sp. nov.	**HMAS-L0141604**	China	J.H. Wang & R.D. Liu	**MW344248**				
S. pseudosubtilissima sp. nov.	**HMAS-L0141606**	China	J.H. Wang & R.D. Liu	**MW344249**				
S. pseudosubtilissima sp. nov.	**HMAS-L0141607**	China	J.H. Wang & R.D. Liu	**MW344250**				
S. pseudosubtilissima sp. nov.	**HMAS-L0141608**	China	J.H. Wang & R.D. Liu	**MW344251**				
S. pseudosubtilissima sp. nov.	**HMAS-L0141615**	China	J.H. Wang & R.D. Liu	**MW344252**				
S. pseudosubtilissima sp. nov.	**HMAS-L0141619**	China	J.H. Wang & R.D. Liu	**MW344253**				
S. pseudosubtilissima sp. nov.	**HMAS-L0141620**	China	J.H. Wang & R.D. Liu	**MW344254**				
S. pseudosubtilissima sp. nov.	**HMAS-L0141623**	China	J.H. Wang & R.D. Liu	**MW344255**				
S. pseudosubtilissima sp. nov.	**HMAS-L0141627**	China	J.H. Wang & R.D. Liu	**MW344256**				
S. pycnoradians sp. nov.	**HMAS-L0139611**	Thailand	W.C. Wang	**MW344257**				
S. pycnoradians sp. nov.	**HMAS-L0139615**	Thailand	W.C. Wang	**MW344258**	**MW357068**	**MW375723**	**MW346610**	**MW346621**
S. pycnoradians sp. nov.	**HMAS-L0139617**	Thailand	W.C. Wang	**MW344259**				
S. pycnoradians sp. nov.	**HMAS-L0141583**	Thailand	W.C. Wang	**MW344260**				
S. sinoaustralis	HMAS-L0137203			KX216699				

S. sinoaustralis	HMAS-L0137204			KX216698	MK206249	—	MK273096	MK273124
S. sinoconcreta sp. nov.	**HMAS-L0139630**	China	S.H. Jiang	**MW344261**				
S. sinoconcreta sp. nov.	**HMAS-L0139631**	China	S.H. Jiang	**MW344262**	**MW357069**	—	—	**MW346622**
S. subtilissimoides sp. nov.	**HMAS-L0130556**	China	J.H. Wang & R.D. Liu	**MK206350**				
S. subtilissimoides sp. nov.	**HMAS-L0139253**	China	S.H. Jiang	**MK206352**	**MK206248**	**MK206229**	**MK273095**	**MK273123**
S. subtilissimoides sp. nov.	**HMAS-L0139254**	China	S.H. Jiang	**MK206353**				
S. subtilissimoides sp. nov.	**HMAS-L0139255**	China	S.H. Jiang	**MK206354**	**MW357070**	**MW375724**	**MW346611**	**MW346623**
S. subtilissimoides sp. nov.	**HMAS-L0139256**	China	S.H. Jiang	**MK206355**	**MK206246**	**MK206227**	**MK273093**	**MK273121**
S. subtilissimoides sp. nov.	**HMAS-L0139293**	China	S.H. Jiang	**MK206356**				
S. subtilissimoides sp. nov.	**HMAS-L0141389**	China	J.H. Wang & R.D. Liu	**MK206351**				
S. univelbiserialis	HMAS-L0137656			KY083734				
S. univelbiserialis	HMAS-L0137657			KY083736	MK206243	MK206224	MK273090	MK273118
S. univelbiserialis	HMAS-L0137658			KY083738	MK206245	MK206226	MK273092	MK273120
S. univelbiserialis	HMAS-L0137659			KY083735	MK206242	MK206223	MK273089	MK273117
S. univelbiserialis	HMAS-L0137660			KY083737	MK206244	MK206225	MK273091	MK273119
Swinscowia jamesii	MPN548				JN887404	JN887388	JN887432	—
Tenuitholiascus porinoides	**HMAS-L0139638**	China	S.H. Jiang	**MK206261**	MK206259	MK352441	MK273106	MK273134
Tenuitholiascus porinoides	**HMAS-L0139639**	China	S.H. Jiang	**MK206262**	MK206258	MK352442	MK273105	MK273133
Tenuitholiascus porinoides	**HMAS-L0139640**	China	S.H. Jiang	**MK206263**	MK206260	MK352443	MK273107	MK273135

[1] The new sequences generated in this study were in bold.
