# Supplementary figures and images for: Twelve New Species Reveal Cryptic Diversification in Foliicolous Lichens of Strigula s.lat. (Strigulales, Ascomycota)
Source: J Fungi (Basel). 2021 Dec 21;8(1):2. doi: 10.3390/jof8010002 (PMC8781847; doi:10.3390/jof8010002)

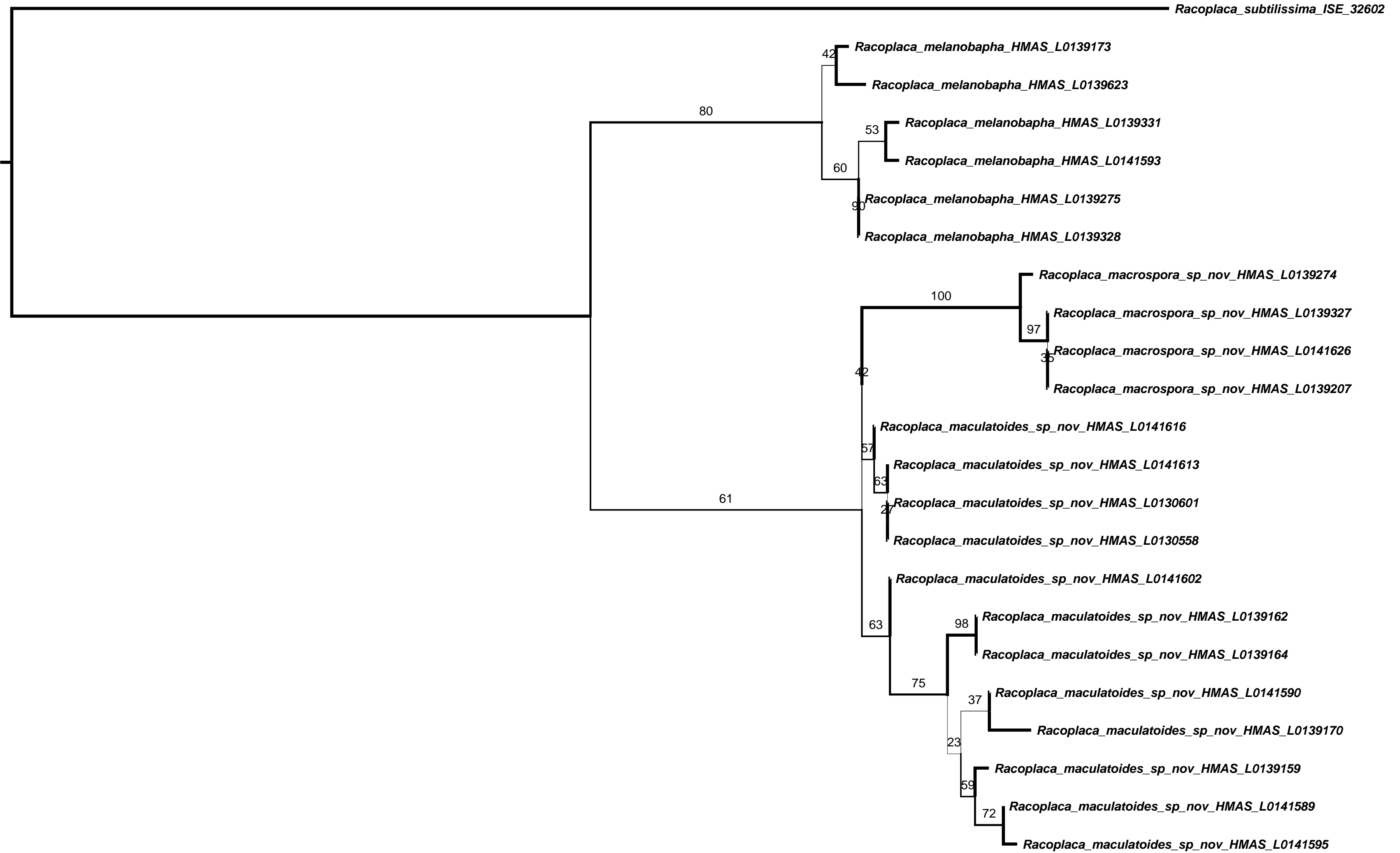

0.03

Supplement: Supplementary file 1 [file jof-08-00002-s001.zip › Figure S1.pdf]
